# Phylogenetic position of *Ligusticopsis* (Apiaceae, Apioideae): evidence from molecular data and carpological characters

**DOI:** 10.1093/aobpla/plac008

**Published:** 2022-03-04

**Authors:** Zi-Xuan Li, Xian-Lin Guo, Megan Price, Song-Dong Zhou, Xing-Jin He

**Affiliations:** 1 Key Laboratory of Bio-Resource and Eco-Environment of Ministry of Education, College of Life Sciences, Sichuan University, Chengdu 610065, Sichuan, P.R. China; 2 Key Laboratory of Conservation Biology on Endangered Wildlife, College of Life Sciences, Sichuan University, Chengdu 610065, Sichuan, P.R. China

**Keywords:** Apioideae, *Ligusticopsis*, *Ligusticum*, mericarp, phylogenetic relationship, plastome

## Abstract

*Ligusticopsis* (Apiaceae, Apioideae) is now considered to have an East-Asia and Sino–Himalaya distribution. The genus was not recognized as a natural and separate genus and was treated as a synonym of *Ligusticum* both in *Flora Reipublicae Popularis Sinicae* and *Flora of China* since first established, though Pimenov *et al.* have made many taxonomic revisions to *Ligusticopsis*, phylogenetic relationships between *Ligusticopsis* and *Ligusticum* have been in dispute. Thirty-four plastomes and 35 nrITS from Apioideae were analysed by RAxML and MrBayes to reconstruct the phylogenetic relationships, along with carpology of 10 species and comparative analyses of 17 plastomes to investigate the evidence supporting the independence of *Ligusticopsis*. As a result, nine species suggested to be *Ligusticopsis* formed a highly supported monophyletic branch (Subclade A) inside Selineae both in maximum likelihood and Bayesian inference; the results of the comparative analyses further supported the monophyly of Subclade A, mainly in the location of genes at the IRa/LSC boundary, the sequence diversity exhibited by various genes (e.g. *trnH-GUG*–*psbA* and *ycf2*) and same codon biases in terminator TAA (relative synonymous codon usage = 1.75). Species in Subclade A also had shared characters in mericarps, combined with other characters of the plant, ‘base clothed in fibrous remnant sheaths, pinnate bracts, pinnate bracteoles longer than rays of umbellule, mericarps strongly compressed dorsally, median and lateral ribs filiform or keeled, marginal ribs winged, and numerous vittae in commissure and each furrow’ should be the most important and diagnostic characters of *Ligusticopsis*. Our phylogenetic trees and other analyses supported the previous taxonomic treatments of Pimenov *et al.* that *Ligusticopsis* should be a natural and separate genus rather than a synonym of *Ligusticum*.

## Teaser

Our paper indicates that *Ligusticopsis*(type species: *Ligusticopsis rechingeriana*) is a natural and separate genus located in tribe Selineae rather than a synonym of genus *Ligusticum*, a result well supported by phylogenetic reconstruction based on nuclear DNA (ITS) and plastome sequences, with further evidence from morphological characters and plastome structure. And the characters from mericarps clearly reveal the morphological divergences between *Ligusticopsis*and the other related genera, which provides effective information for solving the taxonomic problems of the related genera.

## Introduction

**Figure 1.**
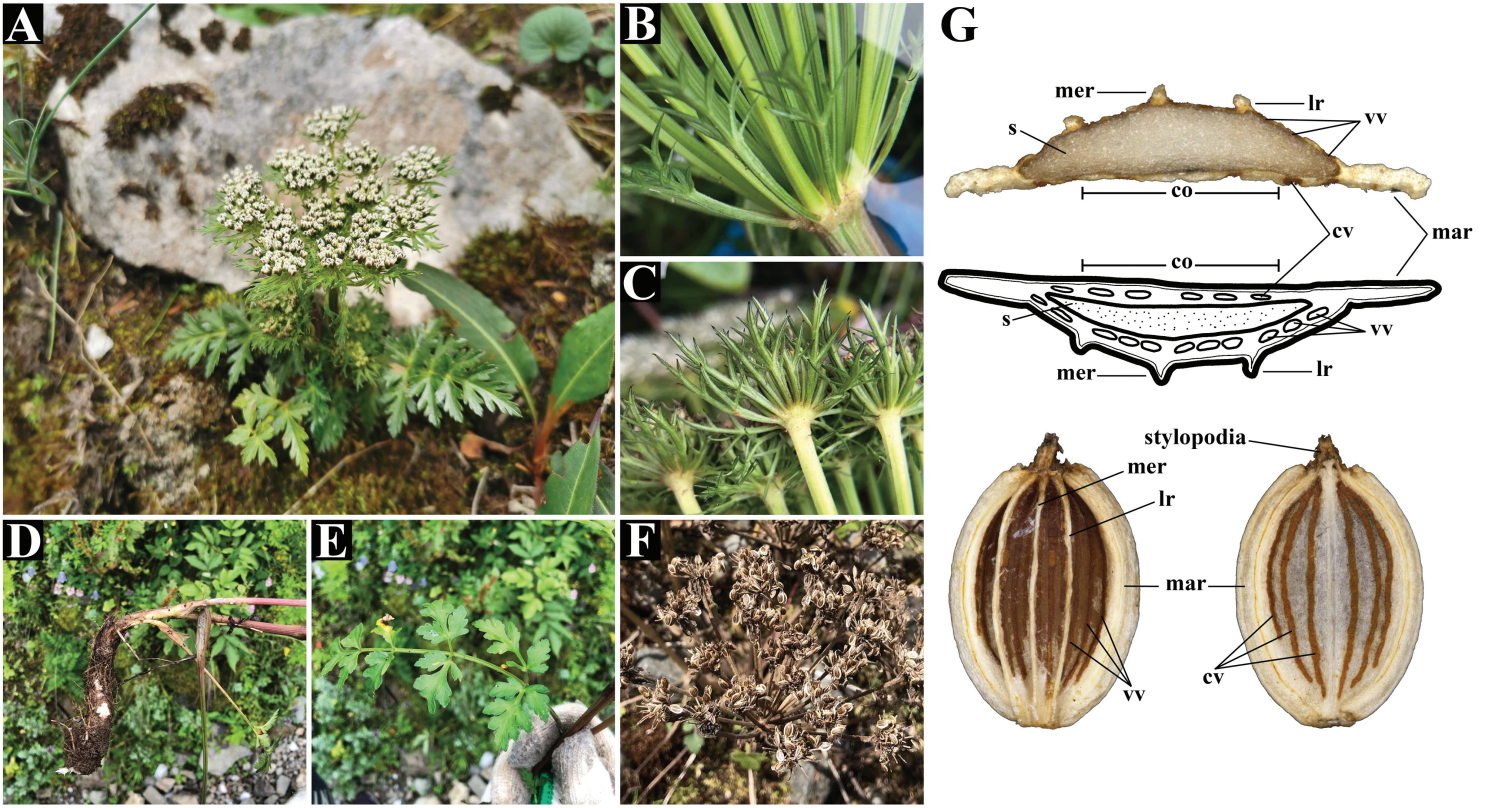
Habit and morphology of *Ligusticopsis rechingeriana*. (A) Habit. (B) Bracts. (C) Bracteoles. (D) Root. (E) Basal leaves. (F) Infructescence. (G) Morphological characters of transverse sections and mericarp, terminologies followed Kljuykov *et al.* (2004), co = commissure; cv = commissural vittae; lr = lateral rib; mar = marginal ribs; mer = median rib; s = seed; vv = vallecular vittae.

*Ligusticopsis* was established by Leute in 1969. By researching the carpology of several *Ligusticum* species from high elevations in China, Leute found that most of these species had mericarp types that clearly corresponded to the ‘Ligusticeen–Typ’ but differed from the known types. The new mericarp type was ‘mericarps strongly compressed dorsally, without mechanical cells arranged in groups or rings’, and these differences in mericarps could be used to distinguish these species from *Selinum* by one vitta per furrow and from *Cortia* by groups of mechanical cells in the rib bases. Leute considered that there was sufficient reason to establish a new genus, and then, the genus *Ligusticopsis*, containing 14 species from China, was established with *Ligusticopsis rechingeriana* (Fig. 1) being selected as the type species. An evolutionary hypothesis for the genus was proposed alongside its classification and concluded that *Ligusticopsis* is a relatively ancient genus (Leute 1969). The main features of the genus *Ligusticopsis* were described by Leute as ‘strongly dorsally flattened mericarps, vallecular vittae numerous, calyx teeth presence’. In 1970, Leute compared *Ligusticum* with the new genus and found that the most important difference between these two was that the genus *Ligusticopsis* has conspicuous calyx teeth (Leute 1970).

The taxonomy of *Ligusticopsis* (Apiaceae subfamily Apioideae) has been controversial as many botanists did not recognize *Ligusticopsis* as a separate genus because of no clear morphological delimitations between *Ligusticopsis* and the nearby genera (Zhou *et al.* 2008a, 2009; Sun and He 2010). Additionally, several species included in *Ligusticopsis* by Leute do not have conspicuous calyx teeth, which blurred the boundaries of the genus (Leute 1970). Studies, including *Flora Reipublicae Popularis Sinicae* and *Flora of China*, have treated *Ligusticopsis* as the synonym of *Ligusticum*, and *Flora of China* treated ‘*Ligusticum* in the broad sense’ (including accepted species in *Flora of China* named by *Ligusticum*) as an artificial assemblage (Hz 1979; Pu 1991; Pu and Watson 2005; Sun *et al.* 2008).

With the progress of molecular phylogeny, a series of studies have shown that neither the genera *Ligusticum* nor *Ligusticopsis* is monophyletic (Downie *et al.* 2010). The latest phylogenetic research using nrDNA sequences (nrITS) has shown that species from genus *Ligusticum* were located in six different clades, while several species that previously belonged to *Ligusticopsis* clustered with other species from nearby genera (e.g. *Cortiella*, *Ligusticum*, *Pachypleurum* and *Selinum*) and formed the ‘Chinese *Ligusticum* Clade’ inside Selineae, but the support between genera and some species was not strong enough to tell from each other. (Valiejo-Roman *et al.* 2006; Zhou *et al.* 2008a, 2009, 2020).

The carpology and mericarp anatomical characters of the taxa from the Apiaceae are of considerable taxonomic and evolutionary importance, which have been increasingly used in conjunction with molecular phylogenetic analyses to indicate stable taxonomy and evolutionary history between species, genera and even within the entire family (Liu *et al.* 2009; Wen *et al.* 2020; Xiao *et al.* 2021). Pimenov *et al.* supported the establishment of *Ligusticopsis* and made several taxonomic revisions of species in *Ligusticopsis* mainly based on carpoanatomical results. *Selinum wallichianum* was placed into *Ligusticopsis* by Pimenov *et al.* (2001), and three species (*Ligusticopsis acuminata*, *Ligusticopsis tenuisecta* and *Ligusticopsis pteridophylla*) previously belonging to *Ligusticopsis* were transferred to *Conioselinum* by Pimenov *et al.* (2003) (Pimenov *et al.* 2001). Recently Pimenov (2017) summarized the nomenclatural combinations of *Ligusticopsis* in China based on reviews of the type specimens and morphological evidence, 18 species (four were newly proposed, and 13 were endemic to China) were recognized, all of which were distributed in the Hengduan Mountains and Qinghai–Tibet Plateau regions of China, while one species published in 2001 was outside China (*L. coniifolia*, West Himalaya, India and Pakistan). And this checklist also recognized that *Ligusticopsis angelicifolia* should be transferred to *Angelica*, while *Ligusticum hispidum* and *Ligusticum involucratum* should be placed into *Ligusticopsis* (Pimenov 2017). Overall, the distribution pattern of these 19 species is from the Hengduan Mountains of China towards the Himalayas (including parts of Qinghai–Tibet Plateau) and up to the western and southern sides (Bhutan, India, Myanmar, Nepal and Pakistan) of the Himalayas.

Several species (e.g. *Ligusticum sinense* in SW China, *Ligusticopsis brachyloba* in Yunnan Province) from ‘*Ligusticum* in the broad sense’ are used as herbs in traditional Chinese medicine, but many are labelled with the same Chinese drug name ‘Gao-Ben’ (Li *et al.* 2001; Pu 1991). These types of herbs are believed to have medicinal value, particularly anthelmintic properties. The extraction of anthelmintic active substances and coumarins has been reported in species transferred into genus *Ligusticopsis* (Kondo *et al.* 2008; Qi *et al.* 2020). Therefore, unclear taxonomy can make scientific investigation of any medical properties difficult. Similarly, the regulated use of Chinese traditional herbs and a search for alternatives can be problematic without clear taxonomy and phylogeny, with molecular authentication of *Ligusticum* species based on nuclear DNA (nrITS2) being reported as a method for determining medically valuable species from other ‘Gao-Ben’ labelled specimens (Liu *et al.* 2019).

In general, clarification of phylogenetic relationships between genera *Ligusticum* and *Ligusticopsis* is necessary. The fact that both *Ligusticopsis* and *Ligusticum* are polyphyletic, and the conflicting views of different botanists on the validity or otherwise of *Ligusticopsis*, implies that the subsumption of *Ligusticopsis* into *Ligusticum* should be approached with caution. Low-resolution phylogenetic relationships based on nuclear gene regions required additional DNA data sets to clarify. Plastomes (i.e. plastid genomes) are one of the good choices, as there have been many reports on the use of plastomes and comparative analyses to construct and resolve the phylogenetic relationships of genera under Apiaceae subfamily Apioideae (Gou *et al.* 2020; Guo *et al.* 2020), while latest study has used plastomes to indicate backbone phylogeny and evolution of subfamily Apioideae with good contributions (Wen *et al.* 2021). What is more, it is reasonable to use the mapping of carpological to phylogenetic results in finding diagnostic characters (Yu *et al.* 2011).

Plastids are important organelles in plants, whose genome DNA (plastome) is usually more conserved than the mitochondrial and nuclear genomes (Marechal and Brisson 2010). Most angiosperm plastomes have a quadripartite circular DNA organization with two copies of inverted repeat (IR) regions, a large single copy (LSC) region and a small single region (SSC) (Wicke *et al.* 2011). Plastomes are maternally inherited, have a high conservation of gene content and genome structure and have more variable sites, making plastomes a significant resource for phylogenetic reconstruction and complicated evolutionary relationships resolution (Ingvarsson *et al.* 2003; Kang *et al.* 2019; Xie *et al.* 2019; Xu *et al.* 2021), and have been widely used for phylogenetic analyses in angiosperms (Jose *et al.* 2015; Wu *et al.* 2020).

In this study, with the addition of seven new plastomes and 11 nrITS sequences, 34 plastomes and 35 nrITS sequences from Apiaceae subfamily Apioideae were analysed, combining phylogenetic results with carpology and comparative analyses of plastomes. We aimed to (i) reconstruct the phylogenetic relationships of *Ligusticopsis* and investigate the rationale for separating *Ligusticopsis* from *Ligusticum*; (ii) indicate evidence from plastomes and carpology in support of phylogenetic relationships and find the diagnostic characters; (iii) investigate the previous taxonomic treatments.

## Materials and Methods

### Taxon sampling

Fresh samples were collected from 11 wild species, being *L. acuminata*, *L. angelicifolia*, *L. brachyloba, L. capillacea*, *L. integrifolia*, *L. modesta*, *L. pteridophylla*, *L. rechingeriana*, *L. tenuisecta*, *L. hispidum* and *S. wallichianum*. Mature and fresh basal leaves were collected from the wild with desiccated and stored in silica gel, respectively. Mericarps from 10 species (*L. angelicifolia*, *L. capillacea*, *L. integrifolia*, *L. modesta*, *L. pteridophylla*, *L. rechingeriana*, *L. tenuisecta*, *L. hispidum*, *L. involucratum* and *S. wallichianum*) were preserved in formaldehyde–acetic acid–alcohol for the following anatomical study. Voucher specimens of these species were deposited in the herbarium of Sichuan University (SZ) **[see****Supporting Information—Table S1****]**.

### DNA extraction, sequencing, assembly and annotation

ITS sequencing was required for the above 11 species. Total genomic DNA was extracted from basal leaf materials using the modified CTAB procedure (Doyle 1987). Amplifications were conducted using 2 μL extracted total genomic DNA, 10 μL ddH_2_O, 1.5 μL of 10 pmol μL^−1^ forward primers, 1.5 μL of 10 pmol μL^−1^ reverse primers and 15 μL *Taq* MasterMix (CWBio, Beijing, China). The nrITS sequences were amplified with primers ITS-4 (5ʹ-TCC TCC GCT TAT TGA TAT GC-3ʹ) and ITS-5 (5ʹ-GGA AGT AAA AGT CGT AAC AAG G-3ʹ) (White *et al.* 1990). Then, PCR cycling profile included a denaturing step at 94 °C for 4 min, followed by 30 cycles of 45 s at 94 °C, annealing at 54 °C for 45 s and extension at 72 °C for 1 min, with a final extension for 10 min at 72 °C. All PCR products were separated using a 1.5 % (w v^−1^) agarose TAE gel and delegated Sangon (Shanghai, China) for sequencing. DNAstar–SeqMan (Burland 2000) was used to edit the newly sequenced DNA and obtain consensus sequences.

Plastomes of seven newly collected species need to be sequenced, which were *L. acuminata*, *L. angelicifolia*, *L. brachyloba*, *L. modesta*, *L. rechingeriana*, *L. tenuisecta* and *S. wallichianum*. We provided 20 µL of total genomic DNA per species for the sequencing process, and total genomic DNA was sequenced using an Illumina Novaseq 6000 platform (Illumina, San Diego, CA, USA) by Personalbio (Shanghai, China). Libraries were constructed with an average length of 350 bp, and the average length of generated reads was 150 bp. FastQ v0.19.7 (Chen *et al.* 2018) was used for quality control of the raw reads, and at least 5 GB clean reads per species were yielded. Then, the remaining clean data were assembled using NOVOPlasty v4.2.1 (Nicolas *et al.* 2020) with *K*-mer 39, where *rbcL* gene of *L. involucratum* (GenBank accession no. NC049054) was used as the seed input. The assembled plastomes were annotated via Plastid Genome Annotator (Qu *et al.* 2019) with the sequences and annotations of *L. involucratum* and *Melanosciadium pimpinelloideum* (GenBank accession no. MN810920) as the reference. The annotation results were then revised manually according to the other plastomes released on GenBank (*L. integrifolia*, NC049055; *L. pteridophylla*, NC049056; *L. hispidum*, NC049053) via Generous v9.0.2 (Drummond 2012). The physical maps of seven new plastomes were generated using OGDraw v1.3.1 (Marc *et al.* 2019).

These seven new annotated plastomes and 11 nrITS sequences data have been submitted to the GenBank under accession numbers MZ491174–MZ491177, and OL547614–OL547616 for plastomes sequences while MZ497218–MZ497222, MZ505394 and OL600820–OL600824 for nrITS sequences. Of 34 plastome sequences and 35 nrITS sequences used in this study, 27 plastome sequences and 31 nrITS sequences were from our lab, and all the sequences are available in GenBank **[see****Supporting Information—Table S1****]**.

### Phylogenetic analyses

The species of *Ligusticopsis* were distributed in Selineae and *Hymenidium* Clade in the previous studies (Valiejo-Roman *et al.* 2006; Zhou *et al.* 2008a, 2020), so species (11 in all) from *Acronema* Clade (two plastomes from *Ligusticum delavayi* and *Meeboldia yunnanensis*), Bupleureae (two plastomes from *Bupleurum*), *Chamaesium* Clade (two plastomes from *Chamaesium*), East-Asia Clade (four plastomes from *Hansenia*) and *Komarovia* Clade (one plastome from *Chuanminshen violaceum*) were selected as composite outgroups to ensure the objectivity of phylogenetic results. The name of the main tribes refers to contributions of Downie *et al.* (2010) and Gou *et al.* (2021).

The gene spacer regions and single-copy coding sequences (CDS) from 34 complete plastomes (10 *Ligusticopsis* plastomes and 24 plastomes from subfamily Apioideae) were extracted and connected, respectively, using Generous v9.0.2 and PhyloSuite v1.2.2. The data sets of plastome CDS, gene spacer regions and 35 nrITS from subfamily Apioideae were used to conduct the phylogenetic analysis, respectively. Data sets were aligned using MAFFT v7.402 (Standley 2013). RAxML v7.2.8 (Stamatakis 2014) with GTR+G was used for maximum likelihood (ML) analyses, and the best-fit model was selected by ModelFinder (Kalyaanamoorthy *et al.* 2017) with 1000 bootstrap replicates. And Bayesian inference (BI) was performed in MrBayes v.3.2.7a (Huelsenbeck 2012) under the GTR+G model. The Markov chain Monte Carlo algorithm was performed for 10 000 000 generations and trees were sampled every 1000 generations for each data partition. The first 20 % of trees were discarded as burn-in and the remaining trees were used to build a 50 % majority-rule consensus tree.

### Comparative plastome analyses

Taxon selection for the comparative analysis was based on phylogenetic results, and a total of 17 plastomes from Selineae and *Hymenidium* Clade were selected, which were 10 *Ligusticopsis* plastomes (*L. acuminata*, *L. angelicifolia*, *L. brachyloba*, *L. capillacea*, *L. integrifolia*, *L. modesta*, *L. pteridophylla*, *L. rechingeriana*, *L. scapiformis* and *L. tenuisecta*), six *Ligusticum* plastomes (*L. hispidum*, *L. involucratum*, *Ligusticum jeholense*, *L. sinense*, *Ligusticum tenuissimum* and *Ligusticum thomsonii*) and *S. wallichianum* plastome.

IRscope (Amiryousefi *et al.* 2018) was used to indicate the boundaries between the IR and SC regions of 17 plastomes, and the results were used as references to visualize the final view after manual revision. Sequence divergence of 17 whole plastomes was performed using the mVISTA (Frazer *et al.* 2004) software in Shuffle-LAGAN mode, with *L. rechingeriana* as the reference, and *L. thomsonii* was chosen as the *X*-axis to indicate sequence divergence globally.

Codon usage analysis was conducted via the program codon W (Peden 2000). To avoid sampling bias (Wright 1990; Yang *et al.* 2018), 53 coding sequences (CDSs) were extracted from these 17 plastomes after removing CDSs less than 300 bp and repeat sequences and then concatenated using PhyloSuite v1.2.2 (Wright 1990; Yang *et al.* 2018; Zhang *et al.* 2020). TBtools (Chen *et al.* 2020) was used to visualize the relative synonymous codon usage (RSCU) (Sharp and Li 1986) values of these 17 plastomes.

### Morphology observations of mericarps

Mericarps of 10 species were photographed using a stereomicroscope (SMZ25, Nikon Corp., Tokyo, Japan). The structures and details for observation were dorsal side views, commissural side views, transverse section views, mericarp shape, calyx teeth, rib shape and vittae. And 10 randomly selected mature mericarps per species for morphological measurements using KaryoType (Altinordu *et al.* 2016). Mericarp terminology followed Kljuykov *et al.* (2004).

## Results

### The plastomes of seven new species

The seven new complete plastome sequences ranged from 148 005 bp (*L. acuminata*) to 163 810 bp (*L. angelicifolia*) in length (Table 1). All seven plastomes showed a typical quadripartite structure **[see****Supporting Information—Figs S1****and****S2****]**, which consisted of a pair of IR regions (17 701–34 723 bp) separated by the LSC (76 900–93 725 bp) and SSC (17 464–18 495 bp) regions. The genome (163 810 bp) and IR region (34 723 bp) of *L. angelicifolia* were the longest of the seven plastomes, but also possessed the shortest LSC region (76 900 bp). The GC content varied across the whole genome, LSC, SSC and IR regions in the seven plastomes, and there was higher GC content (40.8–44.8 %) detected in the IR regions compared to the other regions (LSC, 35.8–36.0 %; SSC, 30.8–31.1 %).

**Table 1.** Comparison of genome content of seven new plastomes. Pseudogenes not included.

Taxon	Length (bp)				GC contents (%)				Number of genes			
	Genome	LSC	SSC	IR	Genome	LSC	SSC	IR	Total	CDS	rRNA	tRNA
*Ligusticopsis acuminata*	148 005	93 314	18 495	17 701	37.6	36.0	31.0	44.8	128	84	8	36
*Ligusticopsis angelicifolia*	163 810	76 900	17 464	34 723	37.4	35.8	31.0	40.8	143	98	8	37
*Ligusticopsis brachyloba*	148 633	92 265	17 588	19 390	37.4	36.0	30.9	43.8	128	84	8	36
*Ligusticopsis modesta*	148 133	92 247	17 568	19 159	37.5	36.0	31.0	44.1	128	84	8	36
*Ligusticopsis rechingeriana*	148 525	91 813	17 654	19 529	37.3	35.9	30.8	43.6	128	84	8	36
*Ligusticopsis tenuisecta*	148 356	93 725	17 653	18 489	37.6	36.0	31.1	44.8	128	84	8	36
*Selinum wallichianum*	148 594	92 281	17 567	19 373	37.4	36.0	31.0	43.8	128	84	8	36

Under the uniform annotation rules, except for *L. angelicifolia*, the remaining six new plastomes contained 128 genes with 84 protein-coding genes (PCGs), 36 transfer RNA genes (tRNAs), eight ribosomal RNA genes (rRNAs) and two genes identified as pseudogenes (*ψycf1* and *ψycf2*). And *L. angelicifolia* plastome contains 143 genes with 98 PCGs, 37 tRNAs, eight rRNAs and one gene identified as a pseudogene (*ψycf1*). The length, GC content and gene components of the seven new plastomes were summarized in Table 1**[see****Supporting Information—Table S2****]**.

### Phylogenetic analyses

**Figure 2.**
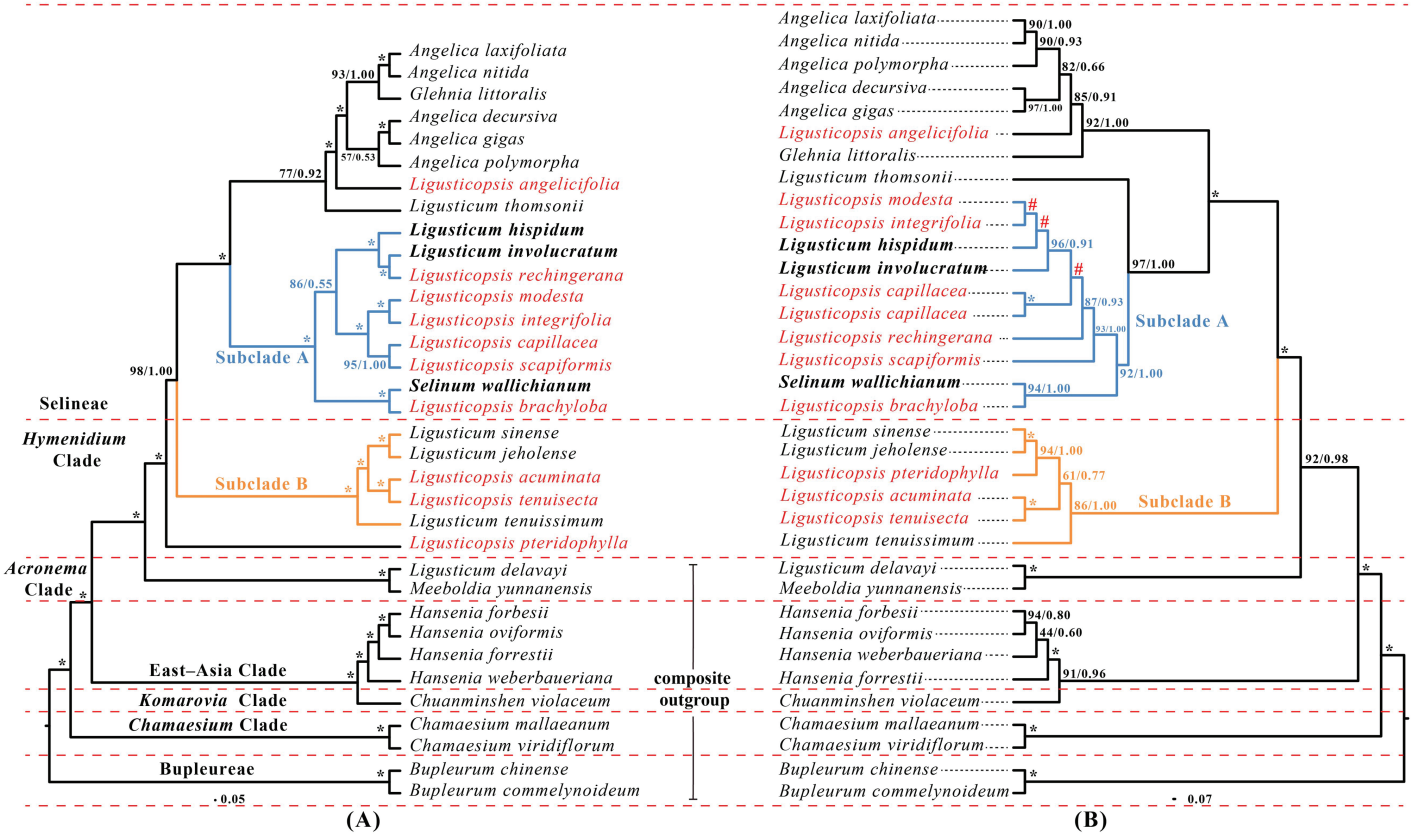
Phylogenetic trees constructed by BI and ML with the posterior probabilities of BI and the bootstrap values of ML above the branches, respectively, (*) represents maximum support in both two analyses, (#) represents those nodes not occurring in the BI strict consensus tree. Species from the treatments by Leute (1969) are named with *Ligusticopsis*, species suggested to be *Ligusticopsis* by Pimenov are marked in bold. (A) Phylogenetic tree of 34 Apioideae taxa based on single-copy CDSs from plastomes. (B) Phylogenetic tree based on 35 nrITS sequences from 34 Apioideae taxa.

Single-copy CDS sequences of 34 plastomes were used to execute the phylogenetic analyses. There were seven major tribes, of which the *Ligusticopsis* species treated by Leute (1969) were non-monophyletic according to ML and BI plastome trees and belonged to Selineae and *Hymenidium* Clade (marked in red, Fig. 2A). Within Selineae, *L. angelicifolia* and *L. thomsonii* clustered with five *Angelica* species and *Glehnia littoralis* to form a clade with moderate support in ML and strong support in BI (BS = 77 %, posterior probabilities [PP] = 0.92). *Ligusticopsis brachyloba*, *L. capillacea*, *L. rechingerana* (type species of *Ligusticopsis*), *L. modesta*, *L. integrifolia* and *L. scapiformis* clustered with *L. involucratum*, *L. hispidum* and *S. wallichianum* forming a branch (Subclade A, Fig. 2A) with strong support (BS = 100 %, PP = 1.00), and all species located in this branch were treated as ‘true *Ligusticopsis*’ by Pimenov. Inside Subclade A, *L. brachyloba* allied with *S. wallichianum* forming a branch (BS = 100 %, PP = 1.00) that was sister to seven other species. *Ligusticopsis rechingerana* was sister to *L. involucratum* and allied with *L. hispidum* forming a branch with strong support (BS = 100 %, PP = 1.00), and this branch was sister to the other four species with moderate support in ML (BS = 72 %) but in BI the support was rather weak (PP = 0.55). *Ligusticopsis capillacea* was sister to *L. scapiformis* and *L. integrifolia* was also sister to *L. modesta*, these two sister branches had strong support (BS = 100 %, PP = 1.00), respectively. We also found that *L. acuminata*, *L. tenuisecta* and *L. pteridophylla* were in *Hymenidium* Clade. *Ligusticopsis acuminata* and *L. tenuisecta* were sister species and clustered with three *Ligusticum* species formed a clade (Subclade B) with strong support (BS = 100 %, PP = 1.00). Inside Subclade B, *L. sinense* and *L. jeholense* were sister species (BS = 100 %, PP = 1.00) and this branch became a sister branch (BS = 100 %, PP = 1.00) to *L. acuminata* and *L. tenuisecta*, while *L. tenuissimum* was sister (BS = 100 %,  PP = 1.00) to the four species above. *Ligusticopsis pteridophylla* belonged to *Hymenidium* Clade in its own branch and it was identified as a sister (BS = 100 %, PP = 1.00) to other species in *Hymenidium* Clade and Selineae. *Ligusticum delavayi* was a sister species to *M. yunnanensis* and located in *Acronema* Clade with strong support (BS = 100 %, PP = 1.00), which was one of the outgroups for *Hymenidium* Clade and Selineae.

A similar topology occurred in the phylogenetic tree based on 35 nrITS from Apiaceae subfamily Apioideae but some species had a different phylogenetic position, and some branches had weak support (Fig. 2B). *Ligusticopsis pteridophylla* was no longer sister to the *Hymenidium* Clade and Selineae but formed a sister branch (BS = 94 %, PP = 1.00) to *L. sinense*, and *L. jeholense* inside the *Hymenidium* Clade (Subclade B, Fig. 2B). *Ligusticum thomsonii* did not cluster with *Angelica* species but was a sister to Subclade A with strong support (BS = 92 %, PP = 1.00). Inside Subclade A, *L. modesta* was still clustered with *L. integrifolia* but with very weak support (BS = 35 %), *L. hispidum* here was a sister to *L. modesta* and *L. integrifolia* but also had weak support (BS = 33 %), and *L. involucratum* was sister to these three species with strong support (BS = 96 %, PP = 0.91). Weak support (BS = 57 %) occurred on the branches between *L. capillacea* and the other four species (*L. involucratum*, *L. hispidum*, *L. modesta* and *L. integrifolia*), and then *L. rechingerana* was sister to these five species with strong support (BS = 87 %, PP = 0.93). *Ligusticopsis scapiformis* was sister to six species above with strong support (BS = 93 %, PP = 1.00).

As for the phylogenetic tree of plastome gene spacer regions, the topological differences were mainly in the positions of *L. thomsonii* and *L. pteridophylla* as well as species within Subclade A **[see****Supporting Information—Fig. S3****]**. *Ligusticum thomsonii* was the sister species to 16 other species from Selineae with strong support (BS = 100 %, PP = 1.00). *Ligusticopsis pteridophylla* was no longer a sister species to Selineae and *Hymenidium* Clade but allied and formed sister branches (BS = 78 %, PP = 0.85) with *L. acuminata*, *L. tenuisecta* and three other *Ligusticum* species inside Subclade B. Within Subclade A, *L. integrifolia* allied with *L*. *scapiformis* and formed a sister branch to *L. modesta* with weak support. The branch including *L. brachyloba* and *S. wallichianum* here was more related to *L. integrifolia*, *L*. *scapiformis*, *L. modesta* and *L. capillacea* with strong support (BS = 88 %, PP = 0.91).

### Comparative plastome analyses

**Figure 3.**
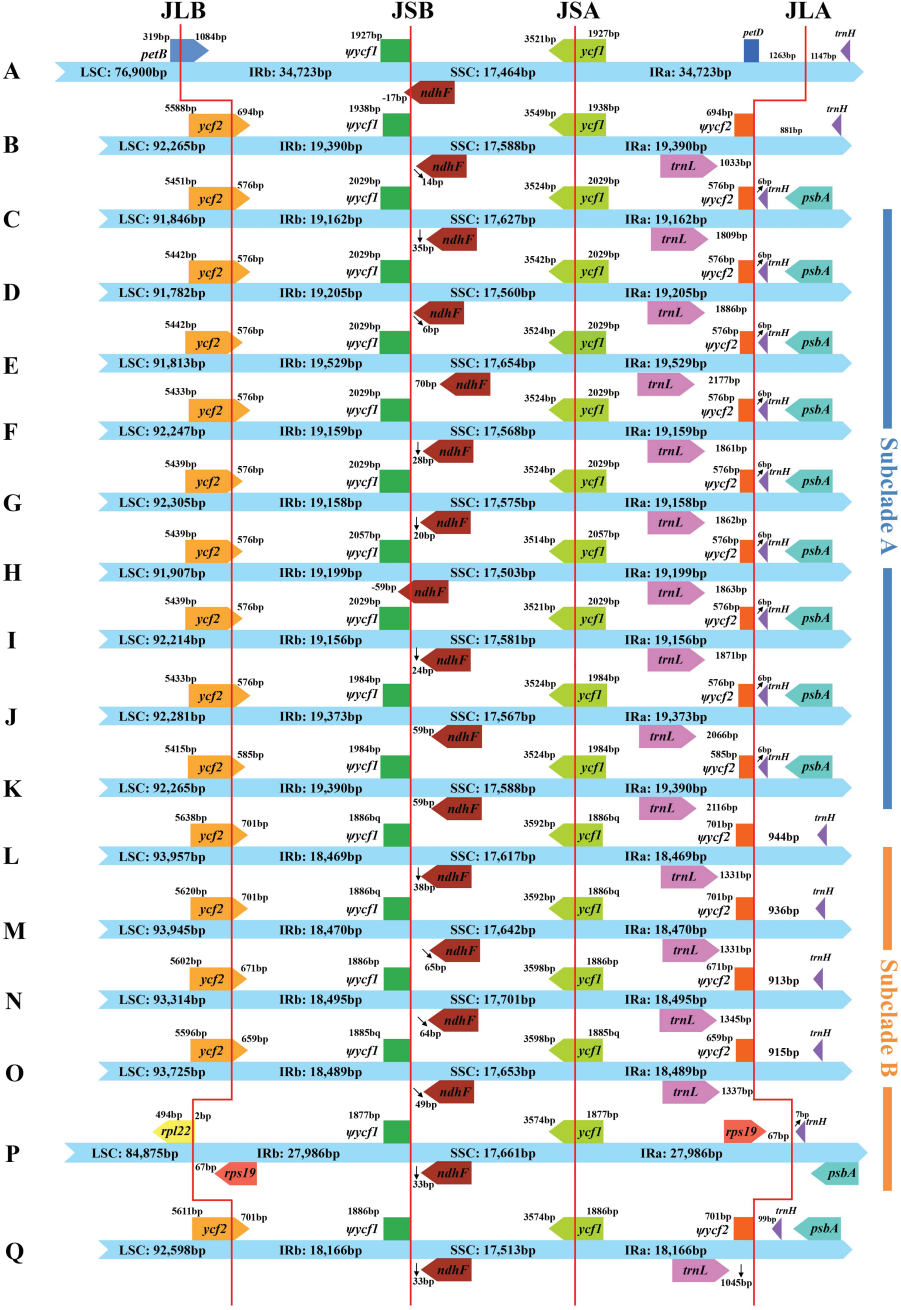
Comparison of LSC, SSC and IR boundary regions between 17 plastomes. Different boxes for genes represent the gene position. JLA: junction of LSC and IRa. JLB: junction of LSC and IRb. JSA: junction of SSC and IRa. JSB: junction of SSC and IRb. (A) *Ligusticopsis angelicifolia*. (B) *Ligusticum thomsonii*. (C) *Ligusticum hispidum*. (D) *Ligusticum involucratum*. (E) *Ligusticopsis rechingeriana*. (F) *Ligusticopsis modesta*. (G) *Ligusticopsis integrifolia*. (H) *Ligusticopsis capillacea*. (I) *Ligusticopsis scapiformis*. (J) *Selinum wallichianum*. (K) *Ligusticopsis brachyloba*. (L) *Ligusticum sinense*. (M) *Ligusticum jeholense*. (N) *Ligusticopsis acuminata*. (O) *Ligusticopsis tenuisecta*. (P) *Ligusticum tenuissimum*. (Q) *Ligusticopsis pteridophylla*.

Seventeen plastomes were compared to observe the gene distribution at their IR boundaries (Fig. 3), while the pseudogenes were visualized with reference to previous studies (Downie and Jansen 2015; Guo *et al.* 2020). The major differences between Subclade A and Subclade B, as well as the other three plastomes, were the gene distribution at IRa/LSC borders (JLA line) and LSC/IRb borders (JLB line). The *trnH* gene region was located near JLA line, and the distance from *trnH* gene to JLA line was the same (6 bp) in Subclade A, while this distance was longer and varied in Subclade B and three other plastomes. Except for *L. angelicifolia* and *L. tenuissimum*, a similar structure was identified across the remaining 15 plastomes at JLB line and JLA line, with some variations. JLB line extended 576–585 bp into the *ycf2* gene in Subclade A, which was shorter than the corresponding region of Subclade B (659–701 bp), *L. pteridophylla* (701 bp) and *L. thomsonii* (694 bp). The distance from *trnL* gene to JLA line in Subclade A was longer (1809–2177 bp) than the corresponding region of Subclade B (1331–1345 bp), *L. pteridophylla* (1045 bp) and *L. thomsonii* (1033 bp). *Ligusticopsis angelicifolia* has the longest IR region such that the IRb region expanded towards the LSC region to the location of the *petB* gene; thus, JLB line extended 1084 bp into *petB* gene. The IRb region of *L. tenuissimum* also showed expansion towards the LSC region, but the JLB line only expands to the location of the *rpl22* gene, and *rpl22* gene spanned JLB line with 2 bp of extensions in IRb region.

**Figure 4.**
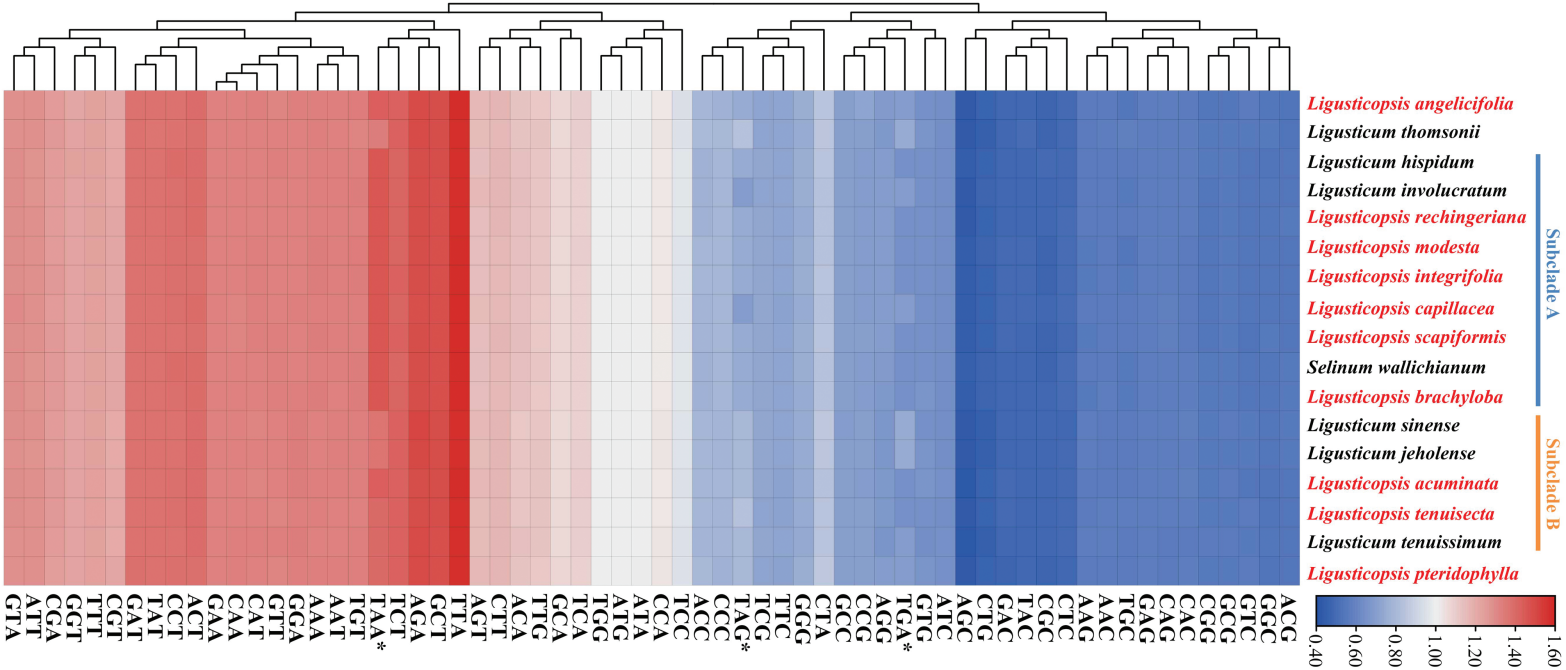
The relative synonymous codon usage (RSCU) values of 53 PCGs for 17 plastomes. (*) to mark the terminator codons.

We extracted 53 CDSs from each of the 17 plastomes for the codon usage analysis. These CDSs encoded 21 585–21 749 codons and leucine (Leu) had the highest number of codons while cysteine (Cys) had the least (Fig. 4). The codons ATG of methionine (Met) and the codons TGG of tryptophan (Trp) had an RSCU value of 1.0 across all plastomes, with 1.0 of RSCU value representing no bias of codon usage. Thirty codons had RSCU values > 1.0 and all had A/T at the third position except TTG of Leu. Among three types of terminator codons, Subclade A had the same codon biases in TAA (RSCU = 1.75), which was higher than Subclade B and the other three plastomes. It was the major difference between Subclade A and other plastomes, while the other two types of terminator codons (TAG and TGA) had RSCU < 1.0 and varied among plastomes. The other 61 types of codons had similar RSCU values in different species (Fig. 4) **[see****Supporting Information—Table S3****]**.

The mVISTA software was used to compare the complete plastomes of 17 species. More sequence conservation occurred in coding regions than non-coding regions. Within Subclade A, there was a high degree of similarity in some highly divergent regions (e.g. *trnH*–*psbA*, *ycf1*, *ycf2*, *rpoC2*, *rpl32*, *psaB*, *ndhF*), but the corresponding regions were significantly different when compared to Subclade B and other three plastomes **[see****Supporting Information—Fig. S4****]**.

### Mericarp morphology

Seven species from Subclade A, being *L. rechingerana*, *L. integrifolia*, *L. modesta*, *L. capillacea*, *L. hispidum*, *L. involucratum* and *S. wallichianum*, had mericarps elliptic to ovate, endosperm commissural face flat, calyx teeth present, filiform or keeled median and lateral ribs, marginal ribs winged, and numerous vittae in commissure (4–8) and each furrow (1–4, not solitary). *Ligusticopsis angelicifolia* from Selineae had elliptic mericarp, endosperm commissural face slightly concave, calyx teeth absent, narrowly winged median and lateral ribs, marginal ribs winged, and numerous vittae in commissure (5–8) and each furrow (2–4). *Ligusticopsis tenuisecta* and *L. pteridophylla* from *Hymenidium* Clade had mericarps slightly concave in seed face, median and lateral ribs winged, but mericarps of these two species have some differences in addition to the characters they share. *Ligusticopsis tenuisecta* had mericarps obcordate and all vittae absent while *L. pteridophylla* had mericarps ovate to orbicular and numerous vittae in commissure (4) and each furrow (2–3) **[see****Supporting Information—Fig. S5****and****Table S4****]**.

## Discussion

In this study, we reconstructed the phylogeny of *Ligusticopsis* and related taxa based on nrITS and plastomes; nine species previously suggested to be ‘true *Ligusticopsis*’ by Pimenov formed a strongly supported monophyletic branch (Subclade A, type species *L. rechingerana* included). The commonalities in carpological characters and the structural consistency in plastomes distinguished from species outside Subclade A and provided further support for the monophyly of the branch. These findings further supported *Ligusticopsis* should be a separate genus in Selineae, and supported the previous taxonomic treatments that *L. hispidum*, *L. involucratum* and *S. wallichianum* should be placed in *Ligusticopsis*, while *L. acuminata*, *L. tenuisecta, L. pteridophylla* and *L*. *angelicifolia* should be transferred out of *Ligusticopsis*.

### Phylogenetic position of *Ligusticopsis* and morphological delimitations between related genera

The results confirmed that nine species (including the type species, i.e. *L. rechingerana*) treated as *Ligusticopsis* by Pimenov (2017) formed a monophyletic branch (i.e. Subclade A) within Selineae. A natural and independent genus should be monophyletic and stable (Funk 1985; Linder and Humphreys 2009; Gomes‐Da‐Silva and Souza‐Chies 2018). Here we agree that treating *Ligusticopsis* as a natural and independent genus within Selineae is justified because (i) the phylogenetic position of the type species (*L. rechingeriana*) is within Selineae, differing from the phylogenetic position (in *Acronema* Clade) of the type species (*Ligusticum scoticum*, i.e. true ‘*Ligusticum*’) of *Ligusticum* (Downie *et al.* 2010; Zhou *et al.* 2020), and (ii) morphological delimitations between *Ligusticopsis* and ‘*Ligusticum* in the broad sense’ objectively exist.

**Figure 5.**
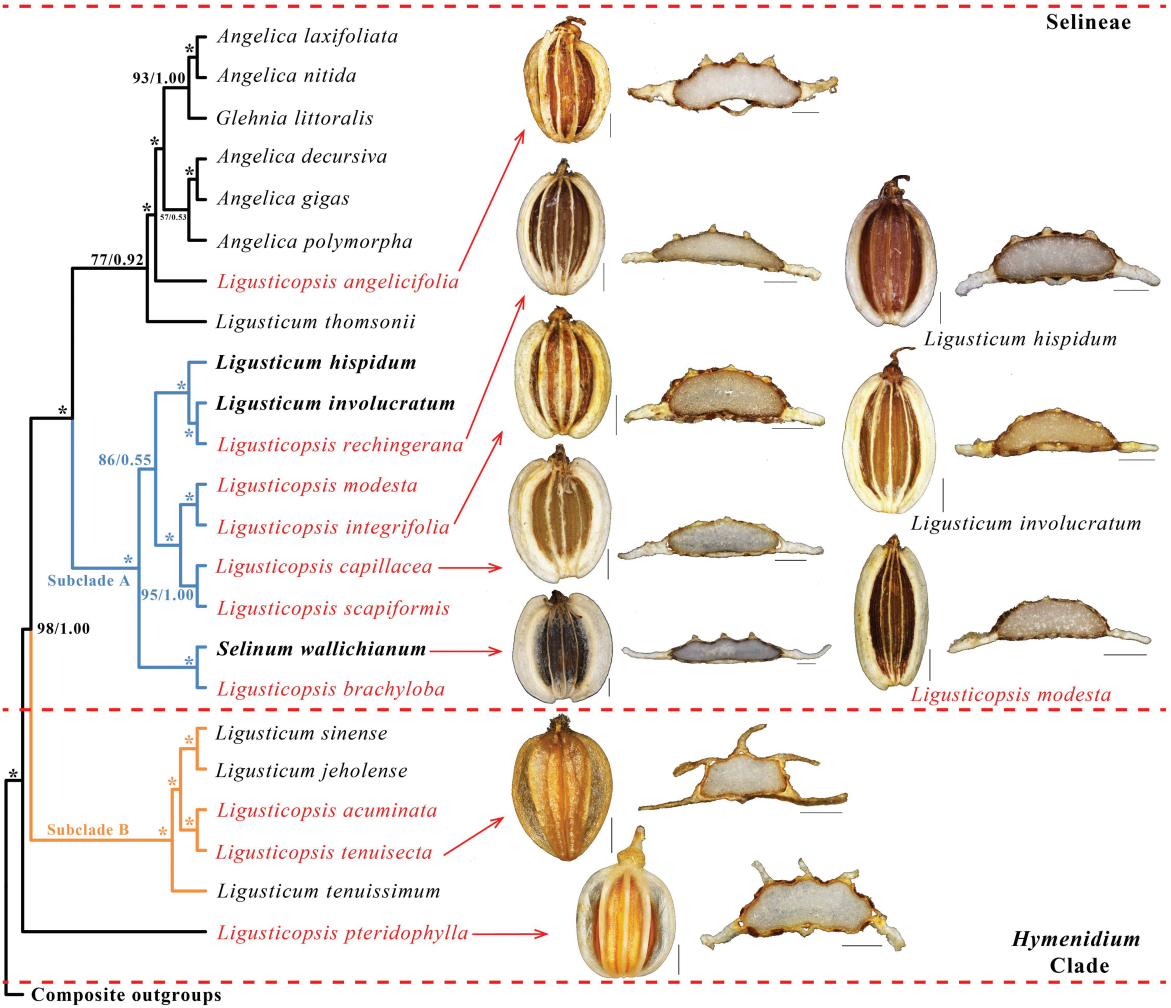
Combination of mericarps and partial plastome CDSs phylogenetic tree from Selineae and *Hymenidium* Clade, with arrows indicating correspondence. Scale bars: dorsal side views = 1 mm, transverse sections = 0.5 mm.

Mapping the mericarps to plastome phylogenetic tree (Fig. 5) and combining other morphological characters **[see****Supporting Information—Table S5****]**, species from Subclade A (i.e. *Ligusticopsis* branch) hold the shared characters ‘base clothed in fibrous remnant sheaths, pinnate bracts, pinnate bracteoles longer than rays of umbellule, subequal rays, obcordate petals, apex notched with incurved apical lobule, calyx teeth presence, mericarps strongly compressed dorsally, median and lateral ribs filiform or keeled, marginal ribs winged, and numerous vittae in commissure (4-8) and each furrow (1–4, not solitary)’. These shared characters can be well distinguished from ‘base without fibrous remnant sheaths, linear bracts and bracteoles, calyx teeth absent, mericarps slightly compressed dorsally’ possessed by Subclade B and *L. pteridophylla*. Shared characters can also be distinguished from ‘base without fibrous remnant sheaths, linear bracteoles, calyx teeth absent, linear purple petals, mericarps slightly compressed dorsally, median and lateral ribs narrowly winged’ possessed by *L*. *angelicifolia* further support for *Ligusticopsis* to be a separate genus. What is more, conspicuous calyx teeth were the main boundary between *Ligusticopsis* and *Ligusticum* according to Leute, but this character was not observed clearly enough on the specimens and fieldwork. Through our research, the combination of mericarp, bract and bracteole morphologies can be more clearly distinguished between *Ligusticopsis* and ‘*Ligusticum* in the broad sense’, which is also consistent with Leute’s view that the differences under genus *Ligusticopsis* come from a combination of a series of characters (Leute 1969). Based on these findings, the morphological delimitations between *Ligusticopsis* and ‘*Ligusticum* in the broad sense’ should be ‘base clothed in fibrous remnant sheaths, pinnate bracts, pinnate bracteoles longer than rays of umbellule, subequal rays, obcordate petals and apex notched with incurved apical lobule, calyx teeth presence, mericarps strongly compressed dorsally, median and lateral ribs filiform, marginal ribs winged, and numerous vittae in commissure (4–8) and each furrow (1–4, not solitary)’.

Zhou *et al.* (2020) determined that species from *Ligusticopsis* were related to *Cortiella*, *Ligusticum*, *Oreocome* and *Selinum* within the ‘Chinese *Ligusticum* Clade’ in Selineae (Zhou *et al.* 2020). However, a limited number of plastomes meant that a comprehensive evaluation of the phylogenetic relationships of these genera was difficult for our research. Based on previous reports on the mericarp morphology of these two genera (*Cortiella* and *Oreocome*), we believe that the mericarp type of *Ligusticopsis* is not a transitional form of these two genera, which indicates the objective existence of morphological divergence between *Ligusticopsis* and these two genera. The mericarp of *Oreocome candollei* (the type species of *Oreocome*) was described as median and lateral ribs all broadly winged and narrow mericarp commissure. And the mericarp of *Cortiella hookeri* (the type species of *Cortiella*) was described as dorsal (median and lateral) ribs broadly winged, often convoluted and crowded when mature, and had vittae two in commissure and one in each furrow, while the most unusual external characters of *C. hookeri* were ‘acaulescent caulescent, forming compact rosettes closely appressed to soil surface’. The morphology described above can be clearly distinguished from *Ligusticopsis*, and the monophyletic relationships exhibited by the nrITS sequences in previous studies, we believe, were most likely due to hybridization, as mixed growth of *Ligusticopsis* species (*S. wallichianum*) and *O. candollei* in the same habitat has also been reported (Norman 1937; Pimenov *et al.* 2001).

*Ligusticum delavayi* and *M. yunnanensis* all formed sister species with strong support (BS = 100 %, PP = 1.00) in *Acronema* Clade, occurring to the three different data sets-based phylogenetic trees. Recent studies have reported that *L. delavayi* allied to several genera (e.g. *Meeboldia*, *Pleurospermum*, *Tilingia* and *Rupiphila*) within *Acronema* Clade (Zhou *et al.* 2020; Gou *et al.* 2021), with complex phylogenetic relationships that requiring more extensive sampling as well as more in-depth studies. Therefore, *L. delavayi* was only one of the outgroups and will not be analysed temporarily in this study. More comprehensive studies on *L. delavayi* and *Acronema* Clade are in progress.

### Evidence from comparative analyses to support the monophyly of Subclade A

Consistency in the distribution of genes around JLA line, the same codon usage bias (RSCU = 1.75) in terminator TAA, and consistency in sequence divergence possessed by some regions (e.g. *trnH*–*psbA*, *ycf1*, *ycf2*, *rpoC2*, *rpl32*, *psaB*, *ndhF*), all the consistency possessed by *Ligusticopsis* branch (i.e. Subclade A) were different from Subclade B and *L*. *angelicifolia*. The *trnH*–*psbA* regions are highly variable and have been suggested to be used as universal barcodes for taxonomic identification (Kress and Erickson 2007; Dong *et al.* 2012). In sequence divergence analysis, the high degree of consistency shown within *trnH*–*psbA* regions supported the monophyly of *Ligusticopsis* branch and can be distinguished from Subclade B. This finding was also supported by ‘the same distance (6 bp) from the *trnH* gene to the JLA line within *Ligusticopsis* branch’ from IR boundary analyses, while in Subclade B this distance was much longer (7–944 bp). The location changes of genes *trnL* and *trnH* may be due to longer non-coding region fragments during the evolution history of *Ligusticopsis*; this kind of events (rearrangement for structural, frequent losses, and few gains) for genes of plastomes have been discovered in angiosperms (Millen 2001; Jansen 2011). What is more, the same codon usage bias in terminator TAA was the most significant difference between *Ligusticopsis* branch and Subclade B. All these findings gave support to the monophyly of Subclade A (true *Ligusticopsis*). In addition, the RSCU values of the other two terminator codons were different from each other between species, along with the high diversity of other regions, may provide additional information to clarify interspecific relationships (Dong *et al.* 2012).

### Validated taxonomic treatments from previously contributions

We agree with the treatment by Pimenov *et al*. (2001, 2017) that three species (*L. hispidum*, *L. involucratum* and *S. wallichianum*) should be placed into *Ligusticopsis* (Pimenov *et al.* 2001). These three species clustered with *Ligusticopsis* branch both in plastome and nrITS phylogenetic trees and were consistent with *Ligusticopsis* branch (Subclade A) in the gene locations around JLA line and codon usage bias in TAA (RSCU = 1.75). Although *L. hispidum* held the rays extremely elongated, both it and other two species share characters (e.g. base clothed in fibrous remnant sheaths, pinnate bracteoles longer than rays of umbellule, pinnate bracts, mericarps strongly compressed dorsally, median and lateral ribs filiform or keeled and marginal ribs winged) consistent with *Ligusticopsis*. What is more, Pimenov *et al.* (2001) clearly described that the closest relative to *S. wallichianum* in mericarp anatomy was *L. brachyloba*. We confirmed this finding cause *S. wallichianum* was sister to *L. brachyloba*, and plastomes of these two species had some commonalities in the same length (5508 bp) of *ycf1* gene and same distance (59 bp) from *ndhF* gene to IRb/SSC boundary, while the codon usage of three types of terminators was also the same (RSCU = 1.75 in TAA, RSCU = 0.68 in TAG and RSCU = 0.57 in TGA).

Three species, *L. acuminata*, *L*. *tenuisecta* and *L. pteridophylla*, were transferred into *Conioselinum* back in 2003 by Pimenov *et al*. through mericarp anatomy (Pimenov *et al.* 2003). This treatment was better supported by our results that these three species located outside Subclade A, while there three species have shared characters ‘linear bracts and bracteoles, median and lateral ribs winged, marginal ribs more broadly winged, and calyx teeth obsolete’ against macromorphological features of *Ligusticopsis* branch. The plastomes of these three species were similar to those of *L. jeholense* and *L. sinense* in terms of gene distribution at the IR boundaries and sequence diversity and were therefore distinguished from *Ligusticopsis* branch. *Ligusticopsis pteridophylla* was not a sister to *Hymenidium* Clade and Selineae anymore but allied with *L. sinense*, and *L. jeholense* both in nrITS tree and plastome gene spacer regions tree, while it has been reported to be tetraploid (Zhou *et al.* 2008b), its unique phylogenetic founding suggests that its evolutionary history may have been complex.

### Interspecific relationships within *Ligusticopsis*

Topology incongruence between phylogenetic trees constructed based on three data sets (plastome CDSs, plastome gene spacer regions and nrITS) occurred mainly within the *Ligusticopsis* branch (Subclade A), which led to unsatisfactory interspecific relationships under genus *Ligusticopsis*. Inconsistencies imply that *Ligusticopsis* may have a complex history of evolution and differentiation.

Several plant lineages have been confirmed to hold inconsistencies in phylogenies between nrDNA and plastomes (Alejandro *et al.* 2016), with many contributions indicated different interspecific relationships may be due to hybridization and very different genetic pathways and mutation rates between plastomes and the nuclear DNA (Marechal and Brisson 2010; Pelser *et al.* 2010). And interspecific hybridization may cause chloroplast capture events during the rapid radiation of species (Rieseberg and Soltis 1991; Fehrer *et al.* 2007), which have also been reported in Apiaceae subfamily Apioideae (Wen *et al.* 2021). We believe interspecific hybridization is more reasonable as many *Ligusticopsis* species have very close or even the same localities, which provided geographical conditions for hybridization. Fieldwork and type specimens have also revealed large morphological differences between species within *Ligusticopsis*, including but not limited to the shape of basal leaves, the degree of hispid and the texture of bracteoles and bracts. For example, both *L. modesta* and *L. integrifolia* are in the same alpine meadows of Likiang Snow Range (type locality) and have very different external morphologies **[see****Supporting Information—Table S5****]**, but their mericarps have considerable structural similarity. The hybridization may have caused chloroplast capture events between these two species, which led these two to be sister species in plastome tree, but the influence on nrITS was too weak to well support these two to be sister species in nrITS tree.

In addition, wind dispersal is one of the main pathways for the dispersal of Apiaceae (Wen *et al.* 2020). Mericarps strongly compressed dorsally, with weakened dorsal (median and lateral) ribs and winged marginal ribs are characters further adapting to wind dispersal, which may lead to a Sino–Himalaya distribution (from Hengduan Mountains, through the Qinghai–Tibet Plateau to the west of Himalaya) with high altitude of *Ligusticopsis*, and in the Qinghai–Tibet Plateau regions, natural hybrids have been recovered in many genera (Wu *et al.* 2021). All these imply during the rapid radiation of species, hybridization may mainly cause the topology differences between different data set-based phylogenetic trees but did not have a greater impact on the diversity of evolutionary.

In the future, the expansion of sampling coverage in Sino–Himalaya regions will help to clarify interspecific relationships under *Ligusticopsis*. Sampling and studying populations outside the type locality can also help to clarify interspecific relationships, as has been reported within Apiaceae subfamily Apioideae (Xiao *et al.* 2017). We suggest that attention be paid to transitional morphological characters that may occur in *Ligusticopsis* outside the type localities, which will help to understand the evolutionary history and trends during the rapid radiation of species in the Qinghai–Tibet Plateau.

## Conclusion

In this study, the phylogenetic reconstruction of genus *Ligusticopsis* was undertaken with the addition of seven new plastomes and 11 new nrITS sequences. A total of 34 plastomes and 35 nrITS sequences were used for analyses; we found that the plastome structures and mericarp anatomical morphology of genus *Ligusticopsis* differed significantly from those of the ‘*Ligusticum* in the broad sense’, and that these differences matched well with the two kinds of phylogenetic trees (plastome and nrITS trees). Plastome structure mainly differs in gene distributions nearby IRa/LSC boundary, biases in the usage of the terminator TAA and sequence diversity within several gene regions (*trnH*–*psbA*, *ycf1*, *ycf2*, *rpl32* and *ndhF*). The morphological delimitations between *Ligusticopsis* and ‘*Ligusticum* in the broad sense’ should be ‘base clothed in fibrous remnant sheaths, pinnate bracts, pinnate bracteoles longer than rays of umbellule, obcordate petals and apex notched with incurved apical lobule, calyx teeth presence, mericarps strongly compressed dorsally, median and lateral ribs filiform or keeled, marginal ribs winged, and numerous vittae in commissure (4–8) and each furrow (1–4, not solitary)’. We support the taxonomic treatments by Pimenov that three species (*L. acuminata*, *L*. *tenuisecta* and *L*. *pteridophylla*) should be transferred from *Ligusticopsis* to *Conioselinum* and three species (*L. hispidum*, *L. involucratum* and *S. wallichianum*) should be placed in *Ligusticopsis*. The results of our molecular phylogenetic study show that genus *Ligusticopsis* is a completely independent and natural genus-level taxon, which should be separated from genus *Ligusticum*, and the boundary between genera is clear in morphological characteristics.

## Supporting Information

The following additional information is available in the  online version of this article—

Figure S1. Plastome map of five *Ligusticopsis* and *Selinum wallichianum*. Genes shown outside and inside the black circle are transcribed in the clockwise and counterclockwise directions, respectively. Different colour boxes indicate different functional groups. LSC, large single copy; SSC, small single copy; IR, inverted repeat.

Figure S2. Plastome map of *Ligusticopsis angelicifolia*. Genes shown outside and inside the black circle are transcribed in the clockwise and counterclockwise directions, respectively. Different colour boxes indicate different functional groups. LSC, large single copy; SSC, small single copy; IR, inverted repeat.

Figure S3. Phylogenetic relationships of 34 Apioideae taxa based on gene spacer regions from plastomes. Tree constructed by Bayesian inference (BI) and maximum likelihood (ML) with the posterior probabilities of BI and the bootstrap values of ML above the branches, respectively, (*) represents maximum support in both two analyses. (#) represents those nodes not occurring in the BI strict consensus tree. Species from the treatments by Leute (1969) are marked in red, species suggested to be *Ligusticopsis* by Pimenov are marked in bold.

Figure S4. mVISTA visualization of alignment for 17 plastomes. Ligusticopsis rechingeriana as the reference. Ligusticum thomsonii as the X-axis. (A) Ligusticopsis angelicifolia. (B) Ligusticum hispidum. (C) Ligusticum involucratum. (D) Ligusticopsis rechingeriana. (E) Ligusticopsis modesta. (F) Ligusticopsis integrifolia. (G) Ligusticopsis capillacea. (H) Ligusticopsis scapiformis. (I) Selinum wallichianum. (J) Ligusticopsis brachyloba. (K) Ligusticum sinense. (L) Ligusticum jeholense. (M) Ligusticopsis acuminata. (N) Ligusticopsis tenuisecta. (O) Ligusticum tenuissimum. (P) Ligusticopsis pteridophylla.

Figure S5. Morphological characters of mericarps from 10 species. (A) Dorsal side views of mericarps. (B) Commissural side views of mericarps. (C) Transverse sections. (D) Line drawings of mericarps. Scale bars: A = 1.0 mm; B = 1.0 mm; C = 0.5 mm.

Table S1. Voucher information and GenBank accession numbers of DNA sequences used in this study. Newly sequenced plastomes and ITS are marked in bold.

Table S2. List of genes encoded in *Selinum wallichianum* and six new *Ligusticopsis* plastomes. (a) to show duplicated genes, (b) to show duplicated genes only in *Ligusticopsis angelicifolia*, (*ψ*) shows pseudogenes.

Table S3. Codon usage and relative synonymous codon usage (RSCU) values of 53 protein-coding genes of 17 plastomes.

Table S4. Synopsis of the carpological data of 10 species.

Table S5. Synopsis of the morphological data from 17 species in Selineae and *Hymenidium* Clade involved in this study. Some data from type specimens and *Flora of China*.

plac008_suppl_Supplementary_Material

## Data Availability

The newly generated DNA sequences have been submitted to NCBI (https://www.ncbi.nlm.nih.gov/) and their GenBank accession numbers, as well as the published sequences from NCBI, are shown in **Supporting Information—Table S1**.

## Notes

**Evolution & Diversity. Chief Editor**: Jeremy Beaulieu
