# Supplementary material for: Phylogenetic position of Ligusticopsis (Apiaceae, Apioideae): evidence from molecular data and carpological characters
Source: AoB Plants. 2022 Mar 4;14(2):plac008. doi: 10.1093/aobpla/plac008 (PMC9035215; doi:10.1093/aobpla/plac008)
Supplement: plac008_suppl_Supplementary_Material [file plac008_suppl_supplementary_material.pdf]

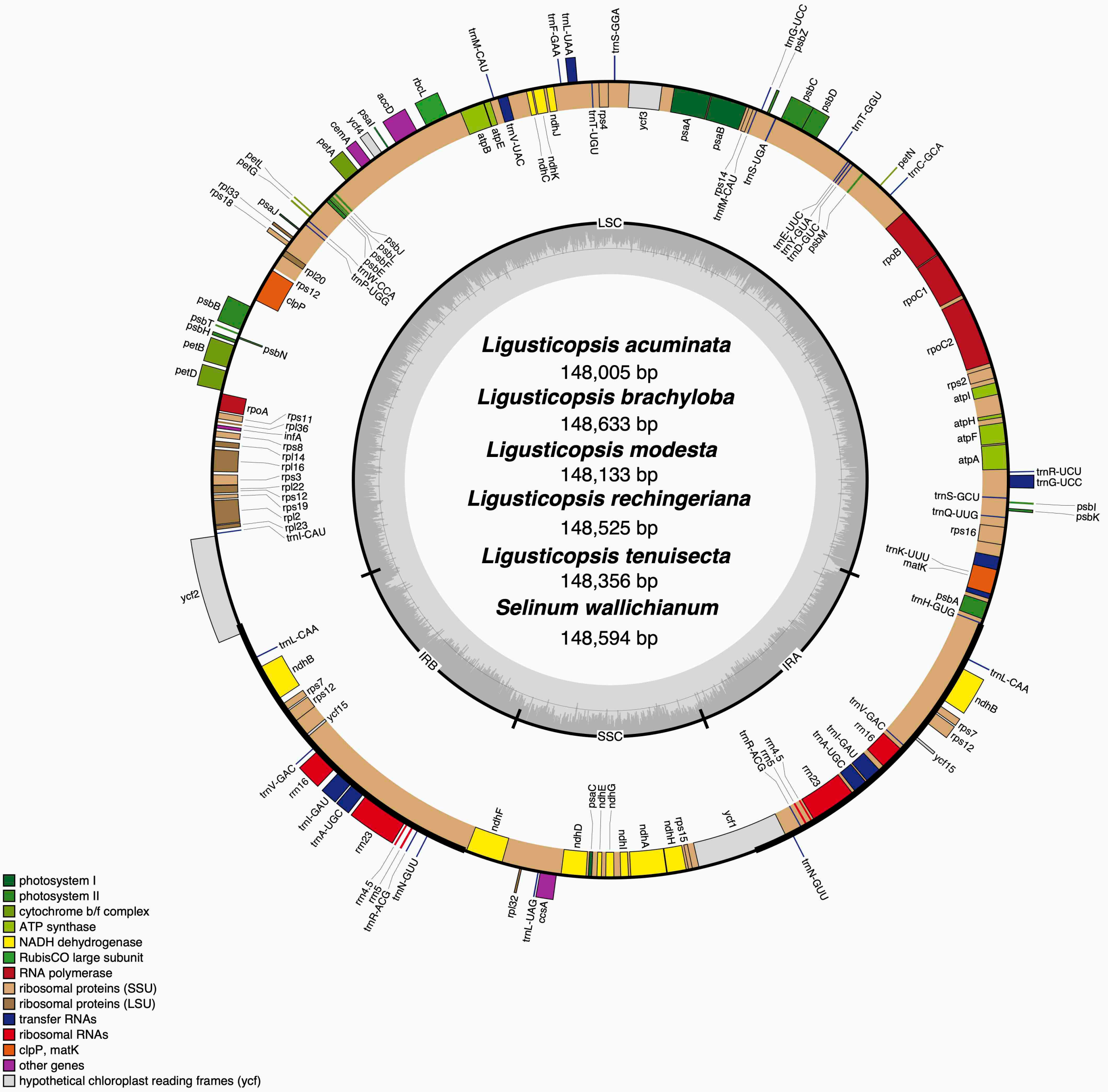

Figure S1. Plastome map of five *Ligusticopsis* and *Selinum wallichianum*.
Genes shown outside and inside the black circle are transcribed in the clockwise and counterclockwise directions, respectively. Different color boxes indicate different functional groups. LSC, large single copy; SSC, small single copy; IR, inverted repeat.

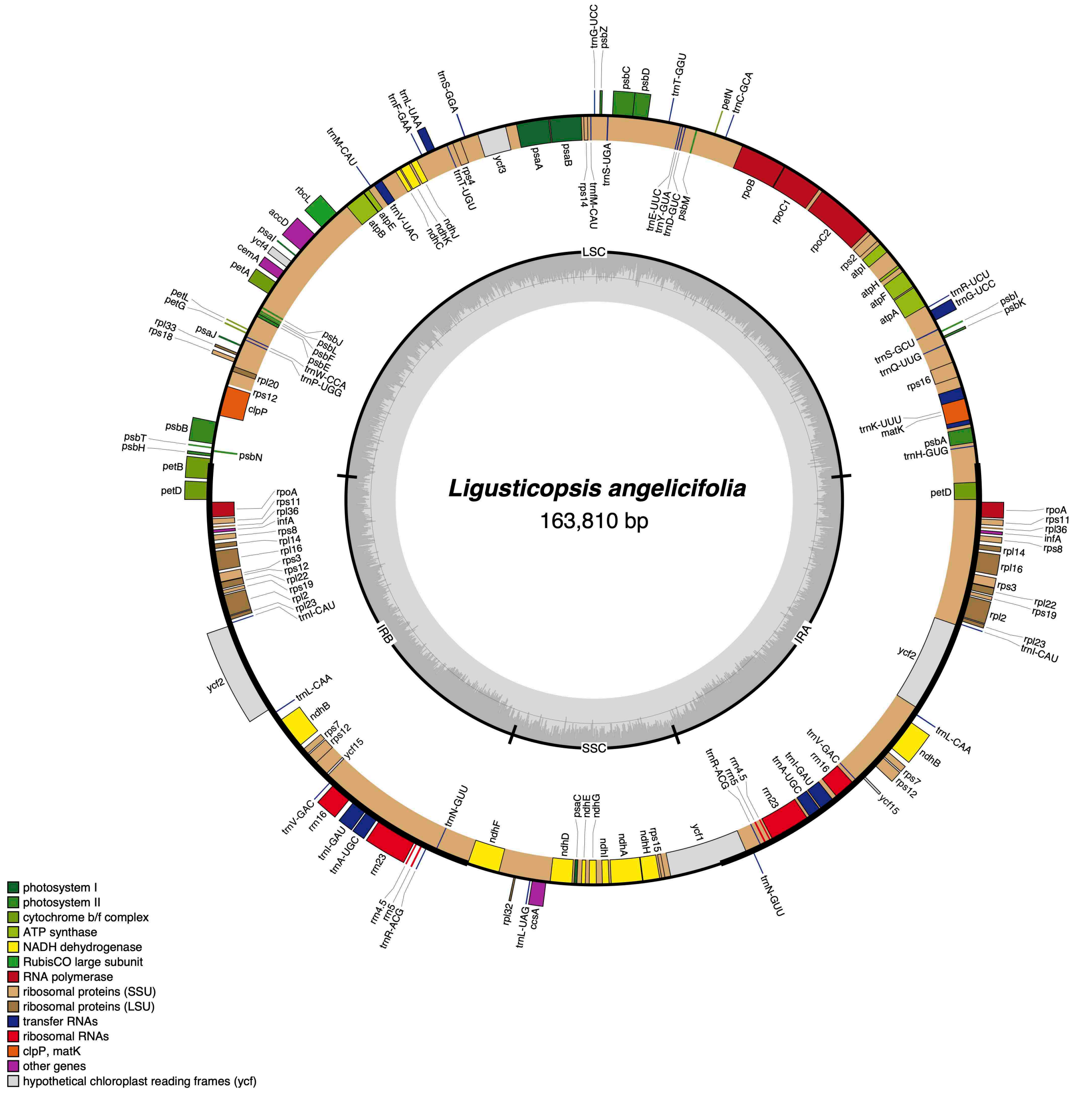

Figure S2. Plastome map of five *Ligusticopsis angelicifolia*.

Genes shown outside and inside the black circle are transcribed in the clockwise and counterclockwise directions, respectively. Different color boxes indicate different functional groups. LSC, large single copy; SSC, small single copy; IR, inverted repeat.

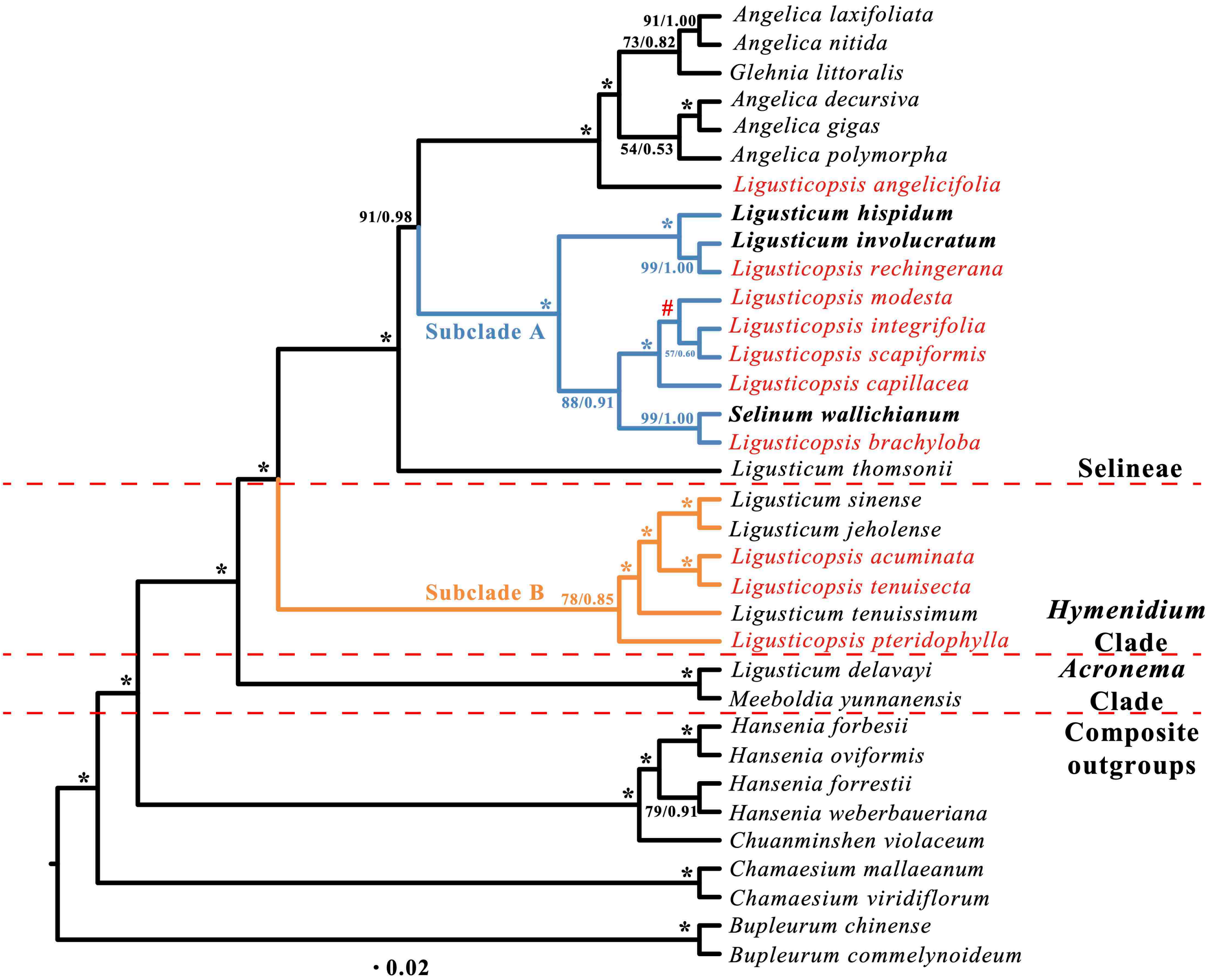

Figure S3. Phylogenetic relationships of 34 Apioideae taxa based on gene spacer regions from plastomes. Tree constructed by Bayesian inference (BI) and maximum likelihood (ML) with the posterior probabilities of BI and the bootstrap values of ML above the branches, respectively, (*) represents maximum support in both two analyses. (#) represents those nodes not occurring in the BI strict consensus tree. Species from the treatments by Leute (1969) are marked in red, species suggested to be *Ligusticopsis* by Pimenov are marked in bold.

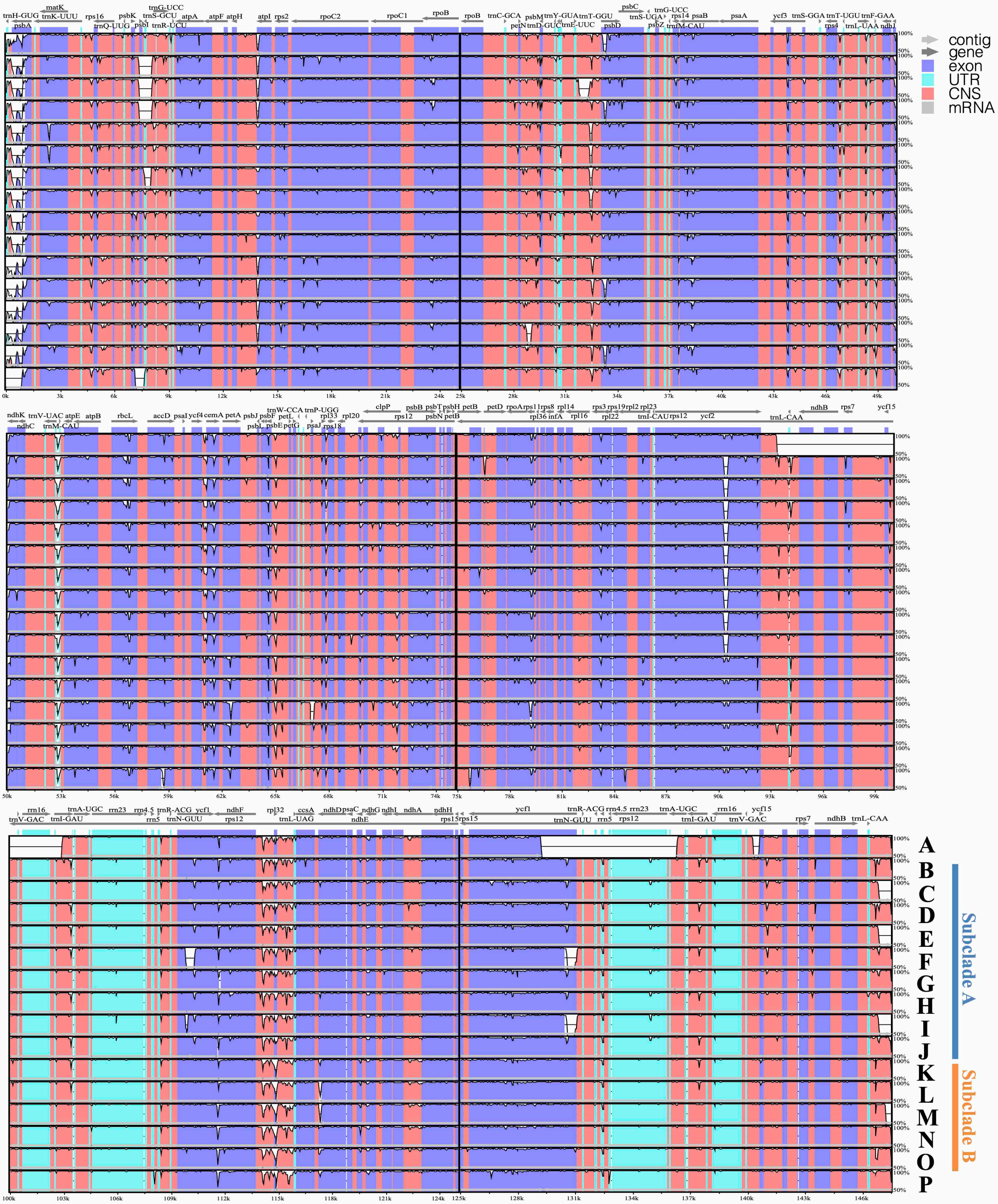

Figure S4. mVISTA visualization of alignment for 17 plastomes. *Ligusticopsis rechingeriana* as the reference. *Ligusticum thomsonii* as the X-axis. (A) *Ligusticopsis angelicifolia*. (B) *Ligusticum hispidum*. (C) *Ligusticum involucratum*. (D) *Ligusticopsis rechingeriana*. (E) *Ligusticopsis modesta*. (F) *Ligusticopsis integrifolia*. (G) *Ligusticopsis capillacea*. (H) *Ligusticopsis scapiformis*. (I) *Selinum wallichianum*. (J) *Ligusticopsis brachyloba*. (K) *Ligusticum sinense*. (L) *Ligusticum jeholense*. (M) *Ligusticopsis acuminata*. (N) *Ligusticopsis tenuisecta*. (O) *Ligusticum tenuissimum*. (P) *Ligusticopsis pteridophylla*.

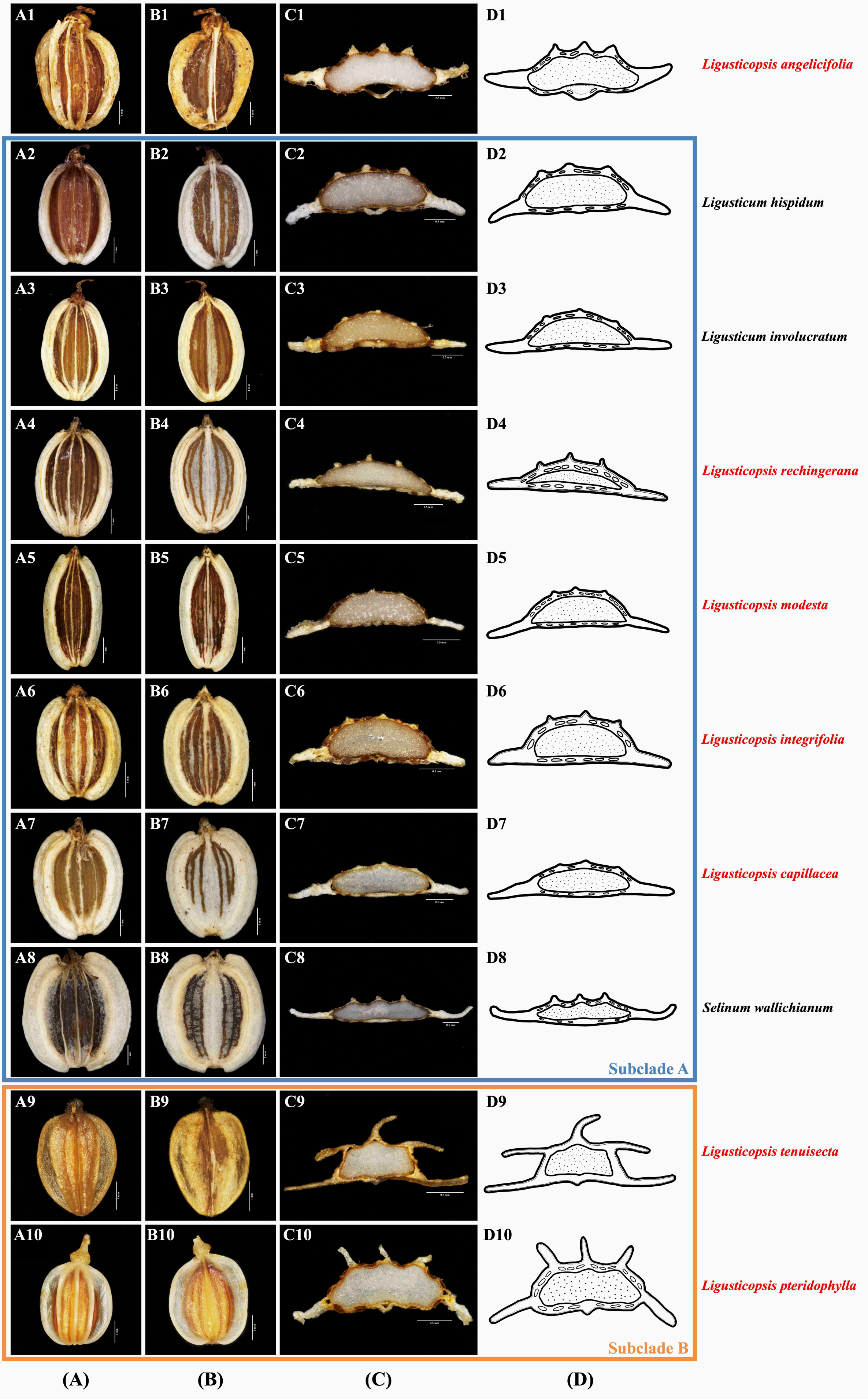

Figure S5. Morphological characters of mericarps from ten species. (A) Dorsal side views of mericarps. (B) Commissural side views of mericarps. (C) Transverse sections. (D) Line drawings of mericarps. Scale bars: A = 1.0mm; B = 1.0mm; C = 0.5mm.

Table S1. Voucher information and GenBank accession numbers of DNA sequences used in this study. Newly sequenced plastomes and ITS are marked in bold.

Taxa	Sequence type	Voucher specimen	Locality	GenBank accession number
Angelica decursiva (Miquel) Franchet & Savatier	ITS	20060825 (SZ)	China, Sichuan,	EU418375
	Plastome	13Q-02-1	Korea, Cheongju-si, Chungcheongbuk-do	KT781591
Angelica gigas Nakai	ITS	SZ744110	Chian, Sichuan, Chengdu	GU395156
	Plastome	13E-39-3	USA, NIH	NC029393
Angelica laxifoliata Diels	ITS	SZ2006071804	China, Yunnan	EU647210
	Plastome	—	China, Sichuan	NC040122
Angelica nitida Wolff	ITS	2006080501 (SZ)	China, Sichuan	EU418378
	Plastome	—	China, Qinghai	MF594405
Angelica polymorpha Maxim.	ITS	SZ744122	Chian, Sichuan	GU395165
	Plastome	KIOM201501014664	Korea, Yuseong-gu	NC041580
Bupleurum chinense DC.	ITS	—	China, Sichuan	EU001334
	Plastome	—	China, Sichuan	MN893666
Bupleurum commelynoideum de Boiss.	ITS	2008082002 (SZ)	China, Sichuan	GU269874
	Plastome	—	China, Sichuan	MT162552
Chamaesium mallaeanum Farille & S. B. Malla	ITS	Strain NLM	China, Xizang	KY744264
	Plastome	—	China, Xizang	MN119369
Chamaesium viridiflorum (Franch.) Wolff ex Shan	ITS	Strain HB	China, Yunnan	KY744260
	Plastome	—	China, Yunnan	MN119373
Chuanminshen violaceum Sheh et Shan	ITS	Isolate cms2	China, Sichuan	HQ185256
	Plastome	—	China, Sichuan	KU921430
Glehnia littoralis Fr. Schmidt ex Miq.	ITS	SZ666775	China, Sichuan	GU395183
	Plastome	—	China, Sichuan	NC034645
Hansenia forbesii (H. Boissieu) Pimenov et Kljuykov	ITS	SZ666939	China, Yunnan	GU390407
	Plastome	—	China, Sichuan	MT834935
Hansenia forrestii (H. Wolff) Pimenov et Kljuykov	ITS	J18192301	China, Sichuan	MN049519
	Plastome	—	China, Sichuan	MT843761
Hansenia oviformis (Shan Renhwa) Pimenov et Kljuykov	ITS	F22	China, Sichuan	MT337430
	Plastome	—	China, Sichuan	MT843762
Hansenia weberbaueriana (Fedde ex H. Wolff) Pimenov et Kljuykov	ITS	J18091701	China, Sichuan	MN049520
	Plastome	—	China, Sichuan	MT843763
Ligusticopsis acuminata (Franch.) Leute	ITS	**L080601 (SZ)**	**China, Sichuan, Maerkang**	**MZ497221**
	Plastome	**L080601 (SZ)**	**China, Sichuan, Maerkang**	**MZ491176**
Ligusticopsis angelicifolia (Franch.) Leute	ITS	**L08110701 (SZ)**	**China, Yunnan, Lijiang**	**OL600821**
	Plastome	**L081107 (SZ)**	**China, Yunnan, Lijiang**	**OL547614**
Ligusticopsis brachyloba (Franch.) Leute	ITS	**L0814 (SZ)**	**China, Chongqing, Nanchuan**	**MZ497218**
	Plastome	**L081401 (SZ)**	**China, Chongqing, Nanchuan**	**MZ491174**
Ligusticopsis capillacea (H. Wolff) Leute	ITS	**L082609 (SZ)**	**China, Yunnan, Deqin**	**OL600820**
		XB	China, Yunnan, Deqin	MT974023
	Plastome	RT2019100601 (SZ)	China, Yunnan, Deqin	NC049051

Ligusticopsis integrifolia (H. Wolff) Leute	ITS	**L081003 (SZ)**	**China, Yunnan, Lijiang**	**MZ497219**
	Plastome	RT2019100202 (SZ)	China, Yunnan, Lijiang	NC049055
Ligusticopsis modesta (Diels) Leute	ITS	**L08190301 (SZ)**	**China, Yunnan, Lijiang**	**OL600822**
	Plastome	**L081903 (SZ)**	**China, Yunnan, Lijiang**	**OL547615**
Ligusticopsis pteridophylla (Franch.) Leute	ITS	**L102502 (SZ)**	**China, Yunnan, Dali**	**MZ505394**
	Plastome	RT2019100302 (SZ)	China, Yunnan, Lijiang	NC049056
Ligusticopsis rechingerana Leute	ITS	**L081103 (SZ)**	**China, Yunnan, Lijiang**	**MZ497220**
	Plastome	**L081103 (SZ)**	**China, Yunnan, Lijiang**	**MZ491175**
Ligusticopsis scapiformis (H. Wolff) Leute	ITS	CT9	China, Sichuan, Baiyu	MT974012
	Plastome	RT2019082001 (SZ)	China, Sichuan, Baiyu	NC049057
Ligusticopsis tenuisecta (H. Boissieu) Leute	ITS	**L092301 (SZ)**	**China, Shaanxi, Langao**	**MZ497222**
	Plastome	**L092301 (SZ)**	**China, Shaanxi, Langao**	**MZ491177**
Ligusticum delavayi Franch.	ITS	LJ	China, Yunnan, Lijiang	MT974017
	Plastome	RT2019100301 (SZ)	China, Yunnan, Lijiang	NC049052
Ligusticum hispidum (Franch.) Wolff	ITS	**L08110501 (SZ)**	**China, Yunnan, Lijiang**	**OL600824**
	Plastome	RT2019100201 (SZ)	China, Yunnan, Lijiang	NC049053
Ligusticum involucratum Franch.	ITS	DB14	China, Yunnan, Lijiang	MT974014
	Plastome	PC2018101905 (SZ)	China, Yunnan, Lanping	NC049054
Ligusticum jeholense (Nakai et Kitag.) Nakai et Kitag.	ITS	LGB1	China, Beijing	KJ999437
	Plastome	—	China, Liaoning, Dalian	MT561037
Ligusticum sinense Oliv.	ITS	Ge131139	China, Guangdong, Guangzhou	MH712648
	Plastome	—	China, Sichuan, Chengdu	NC038088
Ligusticum tenuissimum (Nakai) Kitag.	ITS	JKTM-1-000065	Korea, Anyang-myeon, Jangheung-gun	KP058314
	Plastome	13I-08	Korea, Cheongju-si, Chungcheongbuk-do	NC029394
Ligusticum thomsonii C. B. Clarke	ITS	CJ	China, Sichuan, Baiyu	MT974009
	Plastome	RT2019082301 (SZ)	China, Sichuan, Baiyu	NC049058
Meeboldia yunnanensis (H. Wolff) Constance & F. T. Pu	ITS	G18071908	China, Yunnan, Kunming	MN688997
	Plastome	—	China, Yunnan, Lijiang	MK993275
Selinum wallichianum (DC.) Raizada et H. O. Saxena	ITS	**LD08150601 (SZ)**	**China, Xizang, Lazi County**	**OL600823**
	Plastome	**LD081506 (SZ)**	**China, Xizang, Lazi County**	**OL547616**

Table S2. List of genes encoded in *Selinum wallichianum* and six new *Ligusticopsis* plastomes. (a) to show duplicated genes, (b) to show duplicated genes only in *Ligusticopsis angelicifolia* , (ψ) shows pseudogenes.

Group of Genes		Name of Genes
**Self-replication**	Large subunit of ribosomal proteins (LSU)	*rpl2* [b], *rpl14* [b], *rpl16* [b], *rpl20* , *rpl22* [b], *rpl23* [b], *rpl32* , *rpl33* , *rpl36* [b]
	Small subunit of ribosomal proteins (SSU)	*rps2* , *rps3* [b], *rps4* , *rps7* [a], *rps8* [b], *rps11* [b], *rps12* [a], *rps14* , *rps15* , *rps16* , *rps18* , *rps19* [b]
	Ribosomal RNAs	*rrn4.5* [a], *rrn5* [a], *rrn16* [a], *rrn23* [a]
	RNA polymerase	*rpoA* [b], *rpoB* , *rpoC1* , *rpoC2*
	Transfer RNAs	*trnA-UGC* [a], *trnC-GCA, trnD-GUC, trnE-UUC, trnF-GAA, trnfM-CAU, trnG-UCC* [a], *trnH-GUG, trnI-CAU* [b], *trnI-GAU* [a], *trnK-UUU, trnL-CAA* [a], *trnL-UAA, trnL-UAG, trnM-CAU, trnN-GUU* [a], *trnP-UGG, trnQ-UUG, trnR-ACG* [a], *trnR-UCU, trnS-GCU, trnS-GGA, trnS-UGA, trnT-GGU, trnT-UGU, trnV-GAC* [a], *trnV-UAC, trnW-CCA, trnY-GUA*
**Genes for photosynthesis**	ATP synthase	*atpA* , *atpB* , *atpE* , *atpF* , *atpH* , *atpI*
	Cytochrome b/f complex	*petA* , *petB* , *petD* [b], *petG* , *petL* , *petN*
	NADH dehydrogenase	*ndhA* , *ndhB* [a], *ndhC* , *ndhD* , *ndhE* , *ndhF* , *ndhG* , *ndhH* , *ndhI* , *ndhJ* , *ndhK*
	Photosystem I	*psaA* , *psaB* , *psaC* , *psaI* , *psaJ*
	Photosystem II	*psbA* , *psbB* , *psbC* , *psbD* , *psbE* , *psbF* , *psbH* , *psbI* , *psbJ* , *psbK* , *psbL* , *psbM* , *psbN, psbT* , *psbZ*
	Rubisco	*rbcl*
**Other genes**	C-type cytochrome synthesis gene	*ccsA*
	Envelope membrane protein	*cemA*
	Maturase	*matK*
	Protease	*clpP*
	Subunit of Acetyl-CoA-carboxylase	*accD*
	Translational initiation factor	*infA* [b]
	Hypothetical chloroplast reading frames (ycf)	*ycf1* [a] (*ycf1* , ψ*ycf1*), *ycf2* [a] (*ycf2* , ψ*ycf2*), *ycf2* [b], *ycf3* , *ycf4* , *ycf15* [a]
**Total**		130 (144 in *Ligusticopsis angelicifolia*)

Table S3. Codon Usage and Relative Synonymous Codon Usage (RSCU) Values of 53 Protein-coding Genes of 17 Plastomes.

		Ligusticopsis angelicifolia		*Ligusticum thomsonii*		*Ligusticopsis rechingeriana*		*Ligusticopsis scapiformis*		*Ligusticum involucratum*		*Ligusticum hispidum*		*Ligusticopsis brachyloba*		*Ligusticopsis modesta*	
**Amino Acid**	**Codon**	**Number**	**RSCU**	**Number**	**RSCU**	**Number**	**RSCU**	**Number**	**RSCU**	**Number**	**RSCU**	**Number**	**RSCU**	**Number**	**RSCU**	**Number**	**RSCU**
**Phe**	TTT	836	1.35	837	1.35	837	1.35	838	1.35	834	1.35	840	1.35	836	1.35	836	1.35
	TTC	400	0.65	404	0.65	403	0.65	400	0.65	397	0.65	401	0.65	399	0.65	401	0.65
**Leu**	TTA	763	1.99	761	1.98	763	1.98	770	2.00	759	1.98	765	1.98	767	2.00	764	1.99
	TTG	447	1.17	457	1.19	453	1.18	452	1.17	455	1.18	455	1.18	450	1.17	453	1.18
	CTT	483	1.26	478	1.24	481	1.25	480	1.25	479	1.25	482	1.25	477	1.24	477	1.24
	CTC	156	0.41	156	0.41	160	0.42	157	0.41	159	0.41	157	0.41	160	0.42	162	0.42
	CTA	307	0.80	311	0.81	305	0.79	307	0.80	304	0.79	308	0.80	304	0.79	303	0.79
	CTG	144	0.38	142	0.37	148	0.38	147	0.38	149	0.39	148	0.38	148	0.39	149	0.39
**Ile**	ATT	886	1.44	891	1.44	889	1.44	888	1.44	883	1.43	887	1.43	885	1.43	891	1.44
	ATC	346	0.56	347	0.56	349	0.56	347	0.56	345	0.56	347	0.56	348	0.56	347	0.56
	ATA	615	1.00	612	0.99	620	1.00	617	1.00	624	1.01	626	1.01	620	1.00	616	1.00
**Met**	ATG	502	1.00	498	1.00	494	1.00	492	1.00	493	1.00	493	1.00	491	1.00	492	1.00
**Val**	GTT	446	1.50	451	1.51	450	1.51	451	1.52	448	1.51	453	1.52	451	1.52	449	1.51
	GTC	142	0.48	141	0.47	137	0.46	135	0.45	136	0.46	136	0.46	136	0.46	136	0.46
	GTA	428	1.44	432	1.44	432	1.45	433	1.46	430	1.45	433	1.45	430	1.45	432	1.46
	GTG	170	0.57	174	0.58	170	0.57	170	0.57	169	0.57	171	0.57	172	0.58	169	0.57
**Ser**	TCT	453	1.69	454	1.69	447	1.67	450	1.68	445	1.67	449	1.67	449	1.67	448	1.67
	TCC	251	0.93	252	0.94	258	0.96	254	0.95	257	0.96	259	0.96	259	0.96	255	0.95
	TCA	307	1.14	307	1.14	304	1.13	303	1.13	300	1.13	304	1.13	303	1.13	305	1.14
	TCG	176	0.65	176	0.65	177	0.66	177	0.66	174	0.65	176	0.66	178	0.66	176	0.66
	AGT	333	1.24	332	1.23	327	1.22	328	1.22	331	1.24	326	1.21	327	1.22	326	1.22
	AGC	93	0.35	93	0.35	95	0.35	97	0.36	93	0.35	97	0.36	98	0.36	97	0.36
**Pro**	CCT	361	1.60	360	1.59	364	1.61	363	1.60	364	1.61	365	1.62	364	1.61	364	1.61
	CCC	161	0.71	167	0.74	165	0.73	165	0.73	165	0.73	165	0.73	164	0.73	165	0.73
	CCA	236	1.04	238	1.05	234	1.04	234	1.03	234	1.04	234	1.04	234	1.04	236	1.04
	CCG	147	0.65	143	0.63	139	0.62	143	0.63	139	0.62	140	0.62	140	0.62	140	0.62
**Thr**	ACT	459	1.63	459	1.61	462	1.62	457	1.61	457	1.62	463	1.63	462	1.62	457	1.61
	ACC	207	0.73	212	0.75	203	0.71	206	0.72	202	0.71	202	0.71	202	0.71	207	0.73
	ACA	336	1.19	337	1.19	342	1.20	344	1.21	341	1.21	341	1.20	343	1.20	341	1.20
	ACG	127	0.45	129	0.45	133	0.47	130	0.46	131	0.46	132	0.46	134	0.47	131	0.46
**Ala**	GCT	533	1.80	532	1.80	529	1.79	527	1.79	527	1.79	529	1.80	528	1.79	527	1.79
	GCC	185	0.63	187	0.63	186	0.63	186	0.63	187	0.63	185	0.63	185	0.63	186	0.63
	GCA	329	1.11	326	1.10	326	1.11	327	1.11	326	1.11	326	1.11	327	1.11	325	1.10
	GCG	137	0.46	138	0.47	139	0.47	138	0.47	139	0.47	138	0.47	137	0.47	139	0.47
**Tyr**	TAT	672	1.60	661	1.58	675	1.60	673	1.60	679	1.60	677	1.60	674	1.60	673	1.59
	TAC	170	0.40	174	0.42	170	0.40	170	0.40	172	0.40	171	0.40	170	0.40	172	0.41
**His**	CAT	397	1.52	396	1.51	395	1.51	395	1.52	391	1.51	395	1.51	394	1.51	395	1.52
	CAC	126	0.48	128	0.49	127	0.49	126	0.48	128	0.49	127	0.49	128	0.49	125	0.48
**Gln**	CAA	589	1.51	584	1.51	582	1.51	579	1.50	582	1.51	582	1.51	579	1.51	583	1.51
	CAG	190	0.49	191	0.49	188	0.49	191	0.50	188	0.49	187	0.49	188	0.49	188	0.49
**Asn**	AAT	791	1.52	782	1.51	783	1.52	779	1.52	782	1.52	786	1.52	782	1.51	778	1.52
	AAC	252	0.48	253	0.49	246	0.48	247	0.48	246	0.48	247	0.48	251	0.49	249	0.48
**Lys**	AAA	879	1.52	875	1.52	874	1.52	877	1.53	881	1.52	877	1.52	879	1.53	875	1.53
	AAG	274	0.48	279	0.48	274	0.48	271	0.47	275	0.48	277	0.48	273	0.47	272	0.47
**Asp**	GAT	694	1.60	699	1.60	693	1.60	687	1.59	694	1.60	695	1.60	693	1.60	686	1.59
	GAC	174	0.40	173	0.40	173	0.40	176	0.41	173	0.40	174	0.40	174	0.40	176	0.41
**Glu**	GAA	868	1.51	865	1.51	870	1.51	865	1.51	866	1.51	869	1.51	868	1.51	866	1.51
	GAG	278	0.49	281	0.49	280	0.49	281	0.49	281	0.49	281	0.49	280	0.49	281	0.49
**Cys**	TGT	172	1.54	166	1.50	165	1.52	168	1.52	165	1.53	165	1.52	166	1.53	169	1.54
	TGC	52	0.46	55	0.50	52	0.48	53	0.48	51	0.47	52	0.48	51	0.47	51	0.46
**Trp**	TGG	388	1.00	12	0.68	389	1.00	389	1.00	386	1.00	389	1.00	389	1.00	390	1.00
**Arg**	CGT	281	1.33	281	1.32	281	1.33	280	1.33	280	1.32	279	1.32	280	1.33	281	1.33
	CGC	82	0.39	83	0.39	84	0.40	83	0.39	85	0.40	87	0.41	84	0.40	82	0.39
	CGA	294	1.39	296	1.40	292	1.38	295	1.40	293	1.38	291	1.38	296	1.40	293	1.39
	CGG	97	0.46	99	0.47	98	0.46	97	0.46	97	0.46	98	0.47	99	0.47	97	0.46
	AGA	385	1.82	387	1.82	380	1.80	382	1.81	384	1.81	379	1.80	381	1.81	381	1.81
	AGG	129	0.61	127	0.60	130	0.62	130	0.62	131	0.62	130	0.62	126	0.60	130	0.62
**Gly**	GGT	501	1.33	507	1.34	506	1.34	504	1.34	504	1.34	507	1.35	503	1.34	503	1.34
	GGC	175	0.46	176	0.47	174	0.46	175	0.47	174	0.46	171	0.45	174	0.46	175	0.46
	GGA	571	1.52	569	1.51	569	1.51	568	1.51	567	1.51	569	1.51	571	1.52	570	1.51
	GGG	260	0.69	256	0.68	257	0.68	258	0.69	258	0.69	259	0.69	257	0.68	258	0.69
**TER**	TAA	30	1.70	27	1.53	31	1.75	31	1.75	31	1.75	31	1.75	31	1.75	31	1.75
	TAG	12	0.68	14	0.79	12	0.68	12	0.68	11	0.62	12	0.68	12	0.68	12	0.68
	TGA	11	0.62	12	0.68	10	0.57	10	0.57	11	0.62	10	0.57	10	0.57	10	0.57
**In Total**		21696		21720		21681		21665		21642		21707		21671		21654	

	Selinum wallichianum		*Ligusticopsis integrifolia*		*Ligusticopsis capillacea*		*Ligusticopsis acuminata*		*Ligusticopsis pteridophylla*		*Ligusticum jeholense*		*Ligusticum sinense*		*Ligusticopsis tenuisecta*		*Ligusticum tenuissimum*	
**Amino Acid**	**Number**	**RSCU**	**Number**	**RSCU**	**Number**	**RSCU**	**Number**	**RSCU**	**Number**	**RSCU**	**Number**	**RSCU**	**Number**	**RSCU**	**Number**	**RSCU**	**Number**	**RSCU**
**Phe**	835	1.35	837	1.35	837	1.36	848	1.36	848	1.36	845	1.35	848	1.35	850	1.36	842	1.35
	400	0.65	400	0.65	398	0.64	401	0.64	401	0.64	404	0.65	404	0.65	403	0.64	404	0.65
**Leu**	763	1.99	764	1.99	769	2.00	757	1.99	760	1.98	754	1.98	755	1.97	755	1.98	756	1.98
	453	1.18	453	1.18	452	1.17	453	1.19	451	1.17	454	1.19	453	1.18	452	1.19	454	1.19
	478	1.24	476	1.24	478	1.24	476	1.25	482	1.26	477	1.25	480	1.25	474	1.25	477	1.25
	160	0.42	161	0.42	160	0.42	156	0.41	153	0.40	155	0.41	155	0.40	155	0.41	157	0.41
	303	0.79	306	0.80	304	0.79	302	0.79	313	0.82	309	0.81	314	0.82	304	0.80	307	0.80
	149	0.39	148	0.38	148	0.38	143	0.38	144	0.38	141	0.37	142	0.37	143	0.38	141	0.37
**Ile**	884	1.43	888	1.44	888	1.44	893	1.44	893	1.44	888	1.43	891	1.44	889	1.44	889	1.44
	348	0.56	347	0.56	347	0.56	351	0.57	350	0.57	347	0.56	348	0.56	347	0.56	350	0.57
	618	1.00	617	1.00	615	1.00	617	0.99	611	0.99	622	1.00	619	1.00	622	1.00	615	1.00
**Met**	490	1.00	493	1.00	492	1.00	497	1.00	495	1.00	496	1.00	498	1.00	497	1.00	498	1.00
**Val**	451	1.52	449	1.51	450	1.51	447	1.51	445	1.50	448	1.51	449	1.51	444	1.50	450	1.52
	136	0.46	136	0.46	136	0.46	137	0.46	141	0.48	138	0.47	140	0.47	141	0.48	139	0.47
	429	1.45	434	1.46	437	1.47	428	1.45	428	1.44	427	1.44	431	1.45	430	1.45	427	1.44
	170	0.57	170	0.57	169	0.57	171	0.58	172	0.58	172	0.58	170	0.57	170	0.57	167	0.56
**Ser**	446	1.66	451	1.68	449	1.67	453	1.68	455	1.68	452	1.68	454	1.68	454	1.68	454	1.68
	257	0.96	255	0.95	256	0.95	254	0.94	255	0.94	255	0.95	256	0.95	257	0.95	253	0.94
	304	1.13	304	1.13	303	1.13	310	1.15	306	1.13	307	1.14	307	1.13	309	1.14	308	1.14
	178	0.66	176	0.66	176	0.66	177	0.66	176	0.65	177	0.66	178	0.66	176	0.65	179	0.66
	325	1.21	326	1.22	330	1.23	334	1.24	335	1.24	333	1.23	335	1.24	333	1.23	332	1.23
	100	0.37	97	0.36	97	0.36	92	0.34	94	0.35	95	0.35	95	0.35	94	0.35	93	0.34
**Pro**	365	1.62	364	1.61	366	1.61	363	1.61	362	1.61	362	1.60	362	1.60	363	1.61	362	1.59
	164	0.73	165	0.73	166	0.73	164	0.73	159	0.71	162	0.72	162	0.72	162	0.72	164	0.72
	233	1.03	236	1.04	235	1.04	235	1.04	237	1.05	238	1.05	237	1.05	235	1.04	238	1.05
	140	0.62	140	0.62	140	0.62	141	0.62	143	0.63	143	0.63	143	0.63	143	0.63	144	0.63
**Thr**	462	1.62	458	1.61	461	1.62	460	1.63	455	1.61	460	1.63	461	1.63	461	1.63	460	1.63
	202	0.71	206	0.73	204	0.72	205	0.73	208	0.74	205	0.73	206	0.73	206	0.73	204	0.72
	343	1.20	341	1.20	343	1.20	334	1.18	337	1.19	332	1.18	332	1.17	334	1.18	338	1.20
	134	0.47	131	0.46	132	0.46	131	0.46	129	0.46	133	0.47	133	0.47	131	0.46	129	0.46
**Ala**	527	1.79	527	1.79	527	1.79	535	1.81	533	1.79	534	1.81	535	1.81	533	1.80	530	1.79
	185	0.63	186	0.63	186	0.63	185	0.63	188	0.63	185	0.63	185	0.62	185	0.63	187	0.63
	326	1.11	325	1.10	327	1.11	323	1.09	330	1.11	324	1.10	323	1.09	325	1.10	322	1.09
	138	0.47	139	0.47	140	0.47	141	0.48	139	0.47	140	0.47	142	0.48	140	0.47	143	0.48
**Tyr**	673	1.60	672	1.59	674	1.59	675	1.60	672	1.60	669	1.60	671	1.60	672	1.60	669	1.60
	170	0.40	172	0.41	173	0.41	168	0.40	166	0.40	167	0.40	167	0.40	166	0.40	167	0.40
**His**	396	1.51	395	1.52	394	1.52	391	1.50	394	1.51	394	1.51	395	1.50	392	1.51	397	1.52
	128	0.49	125	0.48	125	0.48	130	0.50	129	0.49	129	0.49	130	0.50	128	0.49	126	0.48
**Gln**	583	1.51	582	1.51	579	1.50	582	1.51	588	1.52	584	1.51	585	1.51	583	1.51	583	1.52
	188	0.49	188	0.49	192	0.50	187	0.49	187	0.48	190	0.49	190	0.49	187	0.49	185	0.48
**Asn**	782	1.52	778	1.52	781	1.52	784	1.52	788	1.52	786	1.52	789	1.52	786	1.51	785	1.51
	248	0.48	249	0.48	249	0.48	250	0.48	249	0.48	250	0.48	250	0.48	253	0.49	256	0.49
**Lys**	874	1.52	877	1.53	875	1.52	877	1.52	875	1.52	879	1.51	878	1.51	876	1.52	872	1.51
	273	0.48	271	0.47	273	0.48	278	0.48	276	0.48	284	0.49	285	0.49	276	0.48	280	0.49
**Asp**	690	1.60	687	1.59	688	1.59	695	1.59	702	1.61	699	1.60	702	1.60	696	1.59	694	1.60
	174	0.40	176	0.41	176	0.41	178	0.41	171	0.39	175	0.40	177	0.40	177	0.41	175	0.40
**Glu**	867	1.51	866	1.51	867	1.51	861	1.51	866	1.51	864	1.51	865	1.51	859	1.51	861	1.51
	280	0.49	281	0.49	280	0.49	282	0.49	281	0.49	281	0.49	282	0.49	282	0.49	282	0.49
**Cys**	165	1.53	169	1.53	169	1.52	169	1.52	171	1.52	170	1.52	170	1.52	168	1.51	168	1.52
	51	0.47	52	0.47	53	0.48	53	0.48	54	0.48	53	0.48	54	0.48	54	0.49	53	0.48
**Trp**	388	1.00	390	1.00	390	1.00	388	1.00	391	1.00	390	1.00	390	1.00	389	1.00	389	1.00
**Arg**	280	1.33	281	1.33	281	1.33	278	1.32	280	1.33	277	1.31	277	1.31	278	1.32	277	1.31
	83	0.39	82	0.39	84	0.40	84	0.40	83	0.39	84	0.40	84	0.40	85	0.40	84	0.40
	296	1.40	294	1.40	294	1.39	295	1.40	289	1.37	292	1.39	291	1.38	295	1.40	292	1.39
	99	0.47	97	0.46	98	0.46	99	0.47	98	0.47	98	0.47	98	0.47	99	0.47	102	0.48
	381	1.80	380	1.80	379	1.79	382	1.81	384	1.82	388	1.84	389	1.85	382	1.81	386	1.83
	128	0.61	130	0.62	132	0.62	126	0.60	130	0.62	125	0.59	125	0.59	126	0.60	123	0.58
**Gly**	504	1.34	504	1.34	504	1.34	506	1.34	506	1.34	504	1.34	504	1.34	507	1.34	504	1.34
	173	0.46	175	0.46	175	0.46	170	0.45	170	0.45	172	0.46	172	0.46	170	0.45	173	0.46
	569	1.51	569	1.51	571	1.52	577	1.53	571	1.52	577	1.53	578	1.53	575	1.52	575	1.53
	259	0.69	258	0.69	256	0.68	257	0.68	259	0.69	255	0.68	255	0.68	258	0.68	254	0.67
**TER**	31	1.75	31	1.75	31	1.75	30	1.70	29	1.64	28	1.58	28	1.58	29	1.64	29	1.64
	12	0.68	12	0.68	11	0.62	13	0.74	13	0.74	13	0.74	13	0.74	14	0.79	13	0.74
	10	0.57	10	0.57	11	0.62	10	0.57	11	0.62	12	0.68	12	0.68	10	0.57	11	0.62
**In Total**	21651		21659		21683		21689		21711		21703		21749		21689		21678	

Table S5. Synopsis of the morphological data from 17 species in Selineae and *Hymenidium* Clade involved in this study. Some data from type specimens and *Flora of China* .

	Angelica laxifoliata	***Angelica nitida***	***Glehnia littoralis***	***Angelica decursiva***	***Angelica gigas***	***Angelica polymorpha***
**Mericarp shape**	Elliptic to orbicular	Ovate	Obcordate, hispid	Elliptic	Elliptic	Elliptic
**Calyx teeth**	Obsolete	Obsolete	Linear-lanceolate	Triangular, shorter than stylopodia	Absent	Absent
**Dorsal compression**	Strong	Strong	Strong	Strong	Strong	Strong
**Median rib shape**	Prominent	Prominent	Lignified broad-winged, hispid	Keeled, acute	Keeled, acute	Narrow winged
**Lateral rib shape**	Equalling as median rib	Equalling as median rib	Equalling as median rib	Equalling as median rib	Equalling as median rib	Equalling as median rib
**Marginal ribs shape**	Broad-winged, broader than body	Winged, narrower than the body	Lignified broad-winged, hispid	Thickly narrow-winged	Broad-winged, subequal to body	Broad-winged, broader than body
**Vittae per furrow**	1	1	Absent	1~3	1~2	1
**Commissural vittae**	2	2	Absent	4~6	2~4	2
**Stem (base)**	Without petiole remains	Without petiole remains	Without petiole remains	Without petiole remains, strongly aromatic	Without petiole remains	Without petiole remains
**Bracts**	Purplish, lanceolate, ciliate	Absent	Absent	Purplish, ovate, sheath-like	Saccate, dark purple	Narrow-lanceolate and ciliate
**Bracteoles**	Long-lanceolate, ciliate	Linear, caudate-acuminate	Linear, caudate-acuminate	Linear to lanceolate, green or purple	Dark purple, ovate-lanceolate	Narrow-linear, purplish, ciliate
**Rays**	Unequal, pubescent	Unequal, pubescent	Unequal, pubescent	Subequal, pubescent	Subequal, rays hispidulous	Subequal, rays densely hispidulous
**Petals**	White, obovate	White or yellowish white, rarely purple-red, long-ovate	White, obovate	Dark purple, obovate or ellipsoid-lanceolate, apex incurved but not notched	Dark purple-red, obovate	White, spatulate
**Stylopodia**	Narrow conical	Flat, dark purple	Narrow conical	Flat	Narrow conical	Narrow conical
**Shape of Leaves**	Blade rhombic-triangular	Blade triangular-ovate, leaflets oblong to elliptic, margin crenate, apex obtuse	Blade broad-ovate, ultimate segments oblong to broadly obovate, apex obtuse-rounded	Blade triangular to ovate, leaflets oblong-lanceolate, margin white-cartilaginous and cuspidate-serrate	Blade triangular-ovate, leaflets oblong, margin irregularly coarse-toothed, apex acute	Blade triangular-ovate, leaflets ovate or rhombic-oblong,

	Ligusticopsis angelicifolia	*Ligusticum thomsonii*	*Ligusticum hispidum*	*Ligusticum involucratum*	*Ligusticopsis rechingerana*	*Ligusticopsis modesta*
**Mericarp shape**	Elliptic	Elliptic	Elliptic	Elliptic	Elliptic to ovate	Elliptic to oblong
**Calyx teeth**	Absent	Triangular, shorter than stylopodia	Linear, longer than stylopodia	Acute, shorter than stylopodia	Acute, shorter than stylopodia	Linear, shorter than stylopodia
**Dorsal compression**	Slightly	Not dorsal compression, but slightly compressed laterally	Strong	Strong	Strong	Strong
**Median rib shape**	Narrow winged	Narrow winged	Filiform	Filiform	Filiform	Filiform
**Lateral rib shape**	Equalling as median rib	Equalling as median rib	Equalling as median rib	Equalling as median rib	Equalling as median rib	Equalling as median rib
**Marginal ribs shape**	Broadly winged	Broadly winged	Winged	Winged	Winged	Winged
**Vittae per furrow**	2~4	2~3	1~3	1~3	1~3	3~4
**Commissural vittae**	5~8	4	6	6	6	8
**Stem (base)**	Without petiole remains	Covered with fibrous remains of petioles	Covered with fibrous remains of petioles	Covered with fibrous remains of petioles	Covered with fibrous remains of petioles	Covered with fibrous remains of petioles
**Bracts**	Absent	Linear, margins white membranous	Pinnate, hispid throughout	Pinnate, densely pilose	Pinnate, densely pilose	Pinnate, densely pilose
**Bracteoles**	Shorter than pedicels, linear	Linear, margins white membranous, shorter than pedicels	Pinnate, hispid throughout, longer than pedicels	Pinnate, hispid throughout, longer than pedicels	Pinnate, hispid throughout, longer than pedicels	Pinnate,ciliate, longer than pedicels
**Rays**	Unequal, pubescent	Equal	Extremely elongated, hispid throughout	Equal, hispid throughout	Equal	Equal
**Petals**	Purple, mucronate	White ovate	White purplish obcordate	White purplish obcordate	White purplish obcordate	White purplish obcordate
**Stylopodia**	Conical	Conical	Conical	Conical	Conical	Conical
**Shape of Leaves**	Blade triangular-ovate	Ovate or oblong, margins irregularly serrate to deeply lobed	Lanceolate in outline, ultimate segments linear	Blade oblong-ovate, ultimate segments oblong-ovate	Blade oblong-ovate, ultimate segments ovate	Blade oblong-ovate, ultimate segments linear or lanceolate, hispid

	Ligusticopsis integrifolia	*Ligusticopsis capillacea*	*Ligusticopsis scapiformis*	*Selinum wallichianum*	*Ligusticopsis brachyloba*	*Ligusticum sinense*
**Mericarp shape**	Elliptic to ovate	Ovate	Elliptic to ovate	Elliptic to orbicular	Elliptic	Elliptic to obcordate
**Calyx teeth**	Subulate, shorter than stylopodia	Lanceolate, shorter than stylopodia	Lanceolate, shorter than stylopodia	Linear, longer than stylopodia	Lanceolate, shorter than stylopodia	Absent
**Dorsal compression**	Strong	Strong	Strong	Strong	Strong	Slightly
**Median rib shape**	Filiform	Filiform	Filiform	Keeled	Keeled	Broadly winged
**Lateral rib shape**	Equalling as median rib	Equalling as median rib	Equalling as median rib	Equalling as median rib	Equalling as median rib	Equalling as median rib
**Marginal ribs shape**	Winged	Winged	Winged	Winged	Winged	Broadly winged
**Vittae per furrow**	1~3	1~3	1~4	1~3	2～3	1
**Commissural vittae**	6	6	4~6	4~8	4～6	2
**Stem (base)**	Covered with fibrous remains of petioles	Covered with fibrous remains of petioles	Covered with fibrous remains of petioles	Covered with fibrous remains of petioles	Covered with fibrous remains of petioles	Without petiole remains
**Bracts**	Pinnate and linear coexist	Pinnate, caducous	Pinnate, caducous	Pinnate	Pinnate	Linear
**Bracteoles**	Pinnate,ciliate, longer than pedicels	Pinnate,ciliate, longer than pedicels	Pinnate, longer than pedicels	Pinnate and linear coexist, longer than pedicels, margins white membranous	Pinnate and linear coexist, longer than pedicels, densely pubescent	Linear, shorter than umbellules
**Rays**	Equal	Equal	Equal	Equal	Equal	Subequal
**Petals**	White obcordate	White purplish obcordate	White purplish obcordate	White obcordate	White obcordate	White obcordate
**Stylopodia**	Conical	Conical	Conical	Conical	Conical	Conical
**Shape of Leaves**	Blade oblong-ovate, ultimate segments oblong-ovate or lanceolate	Blade oblong-ovate, ultimate segments linear-lanceolate, ciliate	Blade oblong-lanceolate, ultimate segments linear to lanceolate	Blade broadly ovate, ultimate segments linear	Blade triangular-ovate, ultimate segments linear	Blade triangular-ovate, ultimate segments oblong-ovate, margins irregularly serrate

	Ligusticum jeholense	*Ligusticopsis acuminata*	*Ligusticopsis tenuisecta*	*Ligusticum tenuissimum*	*Ligusticopsis pteridophylla*
**Mericarp shape**	Elliptic to obcordate	Elliptic to obcordate	Elliptic to obcordate	Elliptic to obcordate	Ovate to orbicular
**Calyx teeth**	Absent	Absent	Absent	Absent	Absent
**Dorsal compression**	Slightly	Slightly	Slightly	Slightly	Slightly
**Median rib shape**	Broadly winged	Broadly winged	Broadly winged	Broadly winged	Broadly winged
**Lateral rib shape**	Equalling as median rib	Equalling as median rib	Equalling as median rib	Equalling as median rib	Equalling as median rib
**Marginal ribs shape**	Broadly winged	Broadly winged	Broadly winged	Broadly winged	Broadly winged
**Vittae per furrow**	1	2~4	Absent	1	2~3
**Commissural vittae**	2	6~8	Absent	2	4
**Stem (base)**	Without petiole remains	Without petiole remains	Without petiole remains	Without petiole remains	Without petiole remains
**Bracts**	Linear	Linear	Linear	Linear, white membranous margined, caducous	Linear
**Bracteoles**	Linear, shorter than umbellules	Linear, shorter than umbellules	Linear, shorter than umbellules	Linear, shorter than umbellules, white membranous margined	Linear, shorter than umbellules
**Rays**	Subequal	Subequal	Subequal	Subequal	Subequal
**Petals**	White obcordate	White obcordate	White obcordate	White obcordate	White obcordate
**Stylopodia**	Conical	Conical	Conical	Conical	Conical
**Shape of Leaves**	Blade broad-ovate, ultimate segments ovate	Blade triangular-ovate, ultimate segments subovate, apex acuminate or caudate	Blade triangular, ultimate segments linear	Blade ternate, ultimate segments linear	Blade ovate, ultimate segments obovate or flabelliform
